# Efficacy and Safety of Roxadustat in Patients with CKD: Pooled Analysis by Baseline Inflammation Status

**DOI:** 10.3390/jcm14020303

**Published:** 2025-01-07

**Authors:** Gabriel Choukroun, Frank Strutz, Alexander Harkavyi, Vicki Santos, Alina Jiletcovici, Lucia Del Vecchio

**Affiliations:** 1 Nephrology Dialysis Transplantation Department, CHU Amiens Picardie and Jules Verne University, 80000 Amiens, France; 2 Department of Nephrology, DKD Helios Klinik Wiesbaden, KfH und Nierenzentrum-Rheumatologie, 65191 Wiesbaden, Germany; strutz@nephrologie-wiesbaden.de; 3 Astellas Pharma Europe, Addlestone, Surrey KT15 2NX, UK; alexander.harkavyi@astellas.com; 4 Astellas Pharma, Inc., Northbrook, IL 60062, USA; vicki.santos29721@gmail.com (V.S.); alina.jiletcovici@astellas.com (A.J.); 5 Department of Nephrology and Dialysis, Sant’Anna Hospital, ASST Lariana, 22042 Como, Italy; lucia.delvecchio@asst-lariana.it

**Keywords:** chronic kidney disease, anemia, inflammation, roxadustat, erythropoiesis-stimulating agent

## Abstract

**Background/Objectives**: Inflammation may contribute to hyporesponsiveness to erythropoiesis-stimulating agents (ESAs) and is often present in patients with chronic kidney disease (CKD). Roxadustat is approved in multiple countries for the treatment of anemia of CKD. This pooled analysis evaluated the efficacy and safety of roxadustat in patients with dialysis-dependent (DD) or non-dialysis-dependent (NDD) CKD by inflammation status. **Methods**: Data from five studies comparing roxadustat versus ESAs were pooled by patient populations in this analysis (NDD: DOLOMITES; DD: ROCKIES, SIERRAS, HIMALAYAS, PYRENEES). The mean change from baseline in hemoglobin levels to Weeks 28–52 and mean weekly dose of roxadustat or ESA at Week 24 were assessed by baseline inflammation levels (determined by high-sensitivity C-reactive protein [hsCRP] levels, divided into quintiles). Safety data were summarized descriptively. **Results**: In total, 613 patients with NDD CKD (roxadustat n = 320; ESA n = 293) and 4072 patients with DD CKD (roxadustat n = 2022; ESA n = 2050) were evaluated. Roxadustat increased hemoglobin levels in a manner similar to ESAs, independent of baseline inflammation status. In both the NDD and DD populations, roxadustat doses did not increase at Week 24 in patients with higher hsCRP levels at baseline. Patients with high baseline hsCRP levels required greater ESA doses at Week 24 compared with patients who had lower baseline hsCRP levels in both patient populations. The incidence rates of treatment-emergent adverse events were generally comparable with those of roxadustat and ESA across hsCRP quintiles in both the NDD and DD populations. **Conclusions**: Roxadustat addresses the multiple causes of anemia of CKD, regardless of inflammatory status, without requiring dose increases.

## 1. Introduction

Anemia, a common complication for patients with chronic kidney disease (CKD), is associated with a decreased health-related quality of life, a greater need for blood transfusions, and an increased risk of hospitalization, cardiovascular events, and mortality [1,2]. Anemia’s prevalence increases with CKD severity and a substantial percentage of patients receiving dialysis are anemic [3]. A lack of erythropoietin is not solely responsible for the underlying etiology of anemia of CKD; impaired iron metabolism and inflammation also play a role in the pathophysiology [2]. The current treatment for anemia of CKD often consists of iron supplementation and the use of an erythropoiesis-stimulating agent (ESA) to maintain or raise hemoglobin levels within the target range [4,5]. ESAs are recombinant forms of human erythropoietin, the primary erythropoiesis regulator [6]. These therapeutic interventions do not address the causes of anemia but rather aim to compensate for the decreased level of erythropoietin [4,6] or compensate for functional or absolute iron deficiency. Additionally, several safety concerns and side effects are associated with ESA therapy, including stroke, hypertension, and seizures, especially when used at high doses [4].

Inflammation may lead to a decreased response to ESAs [7,8,9]. Approximately 30–50% of patients with end-stage kidney failure have increased inflammatory markers [8]. In patients with CKD, inflammation can result in anemia through several mechanisms [9]. Hepcidin, a key regulator of iron homeostasis, is secreted more in inflammatory states [7,9]. Increased levels of hepcidin can lead to reduced iron release from stores and iron absorption from the intestine [8,9]. These factors result in insufficient levels of plasma iron, leading to functional iron deficiency and, thus, inefficient erythropoiesis in the context of ESA treatment. Therefore, hepcidin is a key underlying cause of anemia and ESA resistance [8,9].

Patients are classified as ESA-hyporesponsive if there is no increase in hemoglobin concentration after 1 month of ESA treatment, or, if after a patient has had stable doses of ESA, they require two increases in the ESA dose up to 50% beyond the dose at which they had been able to maintain a stable hemoglobin concentration [5]. Depending on definitions, approximately 10% of patients with anemia of CKD experience ESA hyporesponsiveness, with higher ESA doses associated with an increased risk of cardiovascular events and mortality [9,10]. As ESAs are often used to treat anemia of CKD, alternative therapies are needed, especially for patients who experience ESA hyporesponsiveness.

Roxadustat, a hypoxia-inducible factor prolyl hydroxylase inhibitor (HIF PHI), is an oral medication approved to treat anemia of CKD in multiple countries. Hypoxia-inducible factors (HIFs) are transcription factors that regulate the expression of genes involved in iron metabolism and erythropoiesis [2,11]. Roxadustat stabilizes HIF-α [12] and mimics the body’s natural response to hypoxia, thereby stimulating the production of endogenous erythropoietin and increasing transferrin receptor expression and iron utilization, which together result in coordinated erythropoiesis and increased hemoglobin levels [2,4,13]. Inflammation, a potential cause of ESA hyporesponsiveness, leads to increased levels of hepcidin, thereby limiting iron absorption in the intestine and sequestering iron in macrophages [14]. Roxadustat decreases hepcidin levels despite lower erythropoietin plasma levels compared with ESAs, which may be of particular importance in hyporesponsive patients [4,15]. HIF-PHIs, including roxadustat, are an alternative, particularly for patients with inflammation, who are more likely to be ESA-hyporesponsive.

The objective of this pooled analysis was to evaluate the efficacy and safety of roxadustat in patients with non-dialysis-dependent (NDD) or dialysis-dependent (DD) CKD by baseline inflammation status. High-sensitivity C-reactive protein (hsCRP) is a stable, easily measurable, and widely used marker of inflammation [16] and was used to determine baseline inflammation status in this pooled analysis.

## 2. Materials and Methods

### 2.1. Study Design

**Figure 1:**
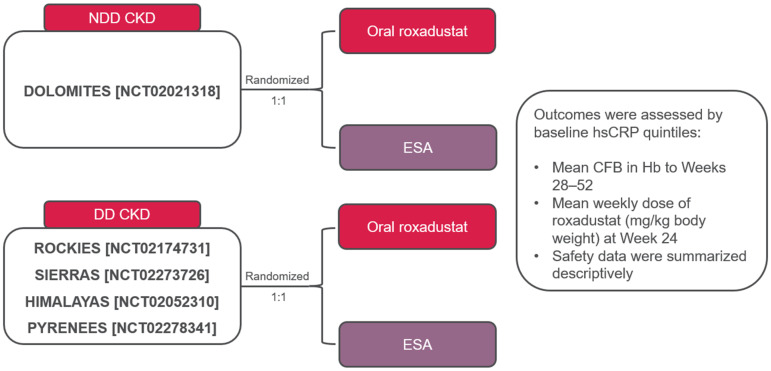
Overview of roxadustat studies in patients with NDD CKD [17] and DD CKD [18,19,20,21]. CFB, change from baseline; CKD, chronic kidney disease; DD, dialysis-dependent; ESA, erythropoiesis-stimulating agent; Hb, hemoglobin; hsCRP, high-sensitivity C-reactive protein; NDD, non-dialysis-dependent.

Five studies were included in this post hoc analysis (Figure 1). Patients with NDD CKD from DOLOMITES were randomized to receive oral roxadustat or an ESA (darbepoetin alfa). The initial roxadustat dose was 70 mg or 100 mg. Patients with DD CKD were randomized 1:1 to receive oral roxadustat (initial dose 70 to 200 mg [ROCKIES, SIERRAS], 100 to 200 mg [PYRENEES], or 70 or 100 mg [HIMALAYAS]) or an ESA (epoetin alfa: ROCKIES, SIERRAS, HIMALAYAS; epoetin alfa or darbepoetin alfa: PYRENEES).

Patients from DOLOMITES (NCT02021318) were analyzed as the NDD population [17]. Patients with DD CKD from four randomized, multicenter, open-label, active-comparator studies (ROCKIES [NCT02174731], SIERRAS [NCT02273726], HIMALAYAS [NCT02052310], PYRENEES [NCT02278341] were pooled, separately from the NDD patient populations, for this analysis [18,19,20,21]. Additional information on target hemoglobin levels are presented in Supplementary Materials.

### 2.2. Patients

Eligible patients in the NDD CKD pooled analysis were adults (≥18 years of age) with anemia of CKD who were not on dialysis and had an estimated glomerular filtration rate (eGFR) of <60 mL/min/1.73 m^2^. In the DOLOMITES study, participants were ineligible if they had received ESA treatment within 12 weeks before randomization, had received a red blood cell transfusion within 8 weeks, had a known hereditary hematologic disease (e.g., thalassemia, sickle cell anemia, pure red cell aplasia, known cause of anemia other than CKD), had a known chronic inflammatory disease that could affect erythropoiesis (e.g., systemic lupus erythematosus, rheumatoid arthritis, celiac disease) even if it was in remission, anticipated undergoing elective surgery expected to lead to significant blood loss, had active or chronic gastrointestinal bleeding, or had received prior treatment with another HIF-PHI or roxadustat.

Eligible patients in the DD CKD pooled analysis were adults (≥18 years of age) with anemia of CKD who were on peritoneal dialysis or hemodialysis, with ferritin levels ≥100 ng/mL and TSAT ≥20%.

Patients were ineligible if they had a recent red blood cell transfusion, had received prior treatment with roxadustat or another HIF-PHI, had active or chronic gastrointestinal bleeding, were anticipating elective surgery with expected blood loss, and/or had a chronic inflammatory disease (such as rheumatoid arthritis, systemic lupus erythematosus, ankylosing spondylitis, psoriatic arthritis, or inflammatory bowel disease).

Prior to enrollment, all patients provided informed written consent. The studies were conducted in accordance with the ethical principles of the Declaration of Helsinki and the International Council for Harmonisation’s Guideline for Good Clinical Practice, and they were reviewed and approved by relevant institutional review boards and/or ethics committees. Individual study details are available at

- https://clinicaltrials.gov/study/NCT02021318 (accessed on 19 December 2024)
- https://clinicaltrials.gov/study/NCT02174731 (accessed on 19 December 2024)
- https://clinicaltrials.gov/study/NCT02273726 (accessed on 19 December 2024)
- https://clinicaltrials.gov/study/NCT02052310 (accessed on 19 December 2024)
- https://clinicaltrials.gov/study/NCT02278341 (accessed on 19 December 2024)

and their associated publications [17,18,19,20,21].

### 2.3. Outcomes

Outcomes were assessed by baseline hsCRP quintile for both the NDD CKD and DD CKD patient populations. The efficacy outcomes assessed were mean change from baseline in hemoglobin levels to Weeks 28–52 and mean weekly dose of roxadustat (mg/kg body weight) or ESA dose (µg/kg for NDD population; mg/kg for DD population) at Week 24. Iron parameter levels were assessed from baseline to Weeks 28–52 (ferritin, serum iron, and TSAT) or Week 24 (hepcidin; DD population only). Safety data, including overall treatment-emergent adverse events (TEAEs) and TEAEs with an incidence of ≥10% in each patient population, were descriptively evaluated using percentages and incidence rates (IRs)/patient exposure years (PEY). Patient follow-up time in PEY for the DD population was defined as

([last dose date − first dose date] + 1)/365.25(1)

IR/100 PEY for the DD population was defined as

100 × number of patients with events/PEY(2)

Patient follow-up time in patient years (PY) for the NDD population was defined as

([first event occurrence or censor date − first dose date + 1)/365.25(3)

IR/100 PY for the NDD population was defined as

100 × number of patients with events/PY(4)

### 2.4. Statistical Analysis

For the NDD population, the change in the hemoglobin level from baseline to Weeks 28–52 by hsCRP quintiles was analyzed by analysis of covariance (ANCOVA; missing at random [MAR]-based multiple imputation model) with the following fixed effect covariates at baseline: hemoglobin and eGFR values in continuous scales, and by treatment group. For patients with DD CKD, the change from baseline hemoglobin levels to Weeks 28–52 by hsCRP quintiles was analyzed by ANCOVA (MAR-based multiple imputation model) with baseline hemoglobin levels as a covariate and cardiovascular/cerebrovascular/thromboembolic history (yes or no), geographical region (US vs. non-US), incident versus stable dialysis (≤4 months vs. >4 months), and treatment group as fixed effects.

For patients with NDD CKD, the mean weekly total roxadustat dose at Week 24 by hsCRP quintiles was analyzed using an ANCOVA model with baseline hemoglobin levels and eGFR as covariates, and quintile rank, treatment group, and history of cardiovascular/cerebrovascular/thromboembolic disease (yes or no) as fixed effects. Adjusted least squares means (LSMs), their differences, and corresponding confidence intervals (CIs) were generated from datasets where missing data were imputed using MAR-based multiple imputation, by treatment group, with baseline hemoglobin levels, baseline eGFR, quintile rank, and history of cardiovascular/cerebrovascular/thromboembolic disease (yes or no) as predictor variables.

For patients with DD CKD, the mean weekly total roxadustat dose at Week 24 by hsCRP quintiles was analyzed with an ANCOVA model with baseline hemoglobin levels as a covariate and study, quintile rank, study-by-quintile rank interaction, region (US, Europe, other), treatment group, and history of cardiovascular/cerebrovascular/thromboembolic disease (yes or no) as fixed effects. Adjusted LSMs, their differences, and corresponding CIs were generated from datasets where missing data were imputed using MAR-based multiple imputation, by treatment group, with baseline hemoglobin levels, quintile rank, geographical region, study, and history of cardiovascular/cerebrovascular/thromboembolic disease (yes or no) as predictor variables.

For patients with NDD CKD, the change from baseline to the mean from Week 28 to Week 52 for each iron parameter (ferritin, serum iron, and TSAT) was analyzed using an ANCOVA model with the following fixed effect covariates: the iron parameter at baseline and baseline eGFR in continuous scales and treatment group. Adjusted LSMs, their difference, and corresponding CIs were generated from datasets where missing data were imputed using MAR-based multiple imputation, by treatment group, with baseline iron parameter and baseline eGFR as predictor variables. For patients with DD CKD, iron parameters were analyzed from baseline to Weeks 28–52 (ferritin, serum iron, and TSAT) or Week 24 (hepcidin) using an ANCOVA model with baseline iron parameter as a covariate and cardiovascular/cerebrovascular/thromboembolic history (yes or no), geographical region (US vs. ex-US), incident vs. stable dialysis (≤4 vs. >4 months), and treatment group as fixed effects. Adjusted LSMs, their difference, and corresponding CIs were generated from datasets where missing data were imputed using MAR-based multiple imputation, by treatment group, with baseline iron parameter, cardiovascular/cerebrovascular/thromboembolic history, geographical region (US vs. ex-US), and incident vs. stable dialysis (≤4 vs. >4 months) as predictor variables.

Clinical characteristics, demographics, TEAEs, and safety data were presented descriptively.

## 3. Results

### 3.1. Demographics and Baseline Disease Characteristics

In total, 613 patients with NDD CKD (roxadustat n = 320; ESA n = 293) were evaluated. The baseline disease characteristics and demographics for patients with NDD CKD treated with roxadustat or ESA are reported in Table S1. At baseline, the mean (SD) hemoglobin (g/dL) levels for roxadustat and ESA were 9.55 (0.75) and 9.55 (0.69), respectively. The mean (SD) hsCRP levels (mg/L) at baseline for roxadustat and ESA were 7.16 (10.61) and 9.49 (21.22), respectively. For patients with NDD CKD, hsCRP levels were divided into quintiles: Q1, ≤0.88 mg/L; Q2, >0.88 mg/L to ≤2.09 mg/L; Q3, >2.09 mg/L to ≤4.39 mg/L; Q4, >4.39 mg/L to ≤11.43 mg/L; Q5, >11.43 mg/L. Overall, a similar percentage of patients in the roxadustat treatment group (56.6%) and ESA treatment group (51.9%) were iron-replete (Table S1). Mean (SD) eGFR (mL/min/1.73 m^2^) levels were similar for roxadustat (20.32 [11.51]) and ESA (20.34 [10.73]; Table S1), regardless of baseline inflammation status (Table S2). Unsurprisingly, patients with a high baseline hsCRP (>11.43 mg/L) were more likely, in general, to have a history of cardiovascular disease than patients with lower baseline hsCRP levels and were more likely to meet the criteria for being iron-depleted (TSAT <20% or ferritin <100 ng/mL; hsCRP >11.43 mg/L: roxadustat: n = 42 [68.9%]; ESA: n = 40 [65.6%]) in both treatment groups (Table S2). Ferritin levels at baseline were generally greater in patients with higher hsCRP levels compared with patients with lower hsCRP levels.

In total, 4072 patients with DD CKD (roxadustat n = 2022; ESA n = 2050) were evaluated (Table S1). The mean (SD) hsCRP levels (mg/L) at baseline for roxadustat and ESA were 9.84 (18.53) and 9.55 (17.82), respectively. For patients with DD CKD, hsCRP levels were divided into quintiles: Q1, ≤1.40 mg/L; Q2, >1.40 mg/L to ≤2.97 mg/L; Q3, >2.97 mg/L to ≤5.98 mg/L; Q4, >5.98 mg/L to ≤13.55 mg/L; Q5, >13.55 mg/L. Few patients met the criteria for iron deficiency (roxadustat: n = 261 [12.9%]; ESA: n = 257 [12.5%]). At baseline, the mean (SD) hemoglobin levels (g/dL) for roxadustat (9.80 [1.29]) and ESA (9.83 [1.29]) were similar, regardless of baseline hsCRP levels (Table S3). In both treatment groups, a higher proportion of patients with baseline hsCRP >5.98 mg/L had a history of cardiovascular disease than those with baseline hsCRP ≤5.98 mg/L, and patients with baseline hsCRP >13.55 mg/L were more likely to meet the criteria for being iron-depleted (TSAT <20% or ferritin <100 ng/mL; hsCRP >13.55 mg/L: roxadustat: n = 81 [20.3%]; ESA: n = 97 [23.4%]) than those with baseline hsCRP ≤13.55 mg/L. Ferritin levels at baseline were higher in patients with hsCRP levels >1.40 mg/L (Q2–Q5) compared with patients with lower hsCRP levels (<1.40 mg/L [Q1]; Table S3).

### 3.2. Efficacy Outcomes

**Figure 2:**
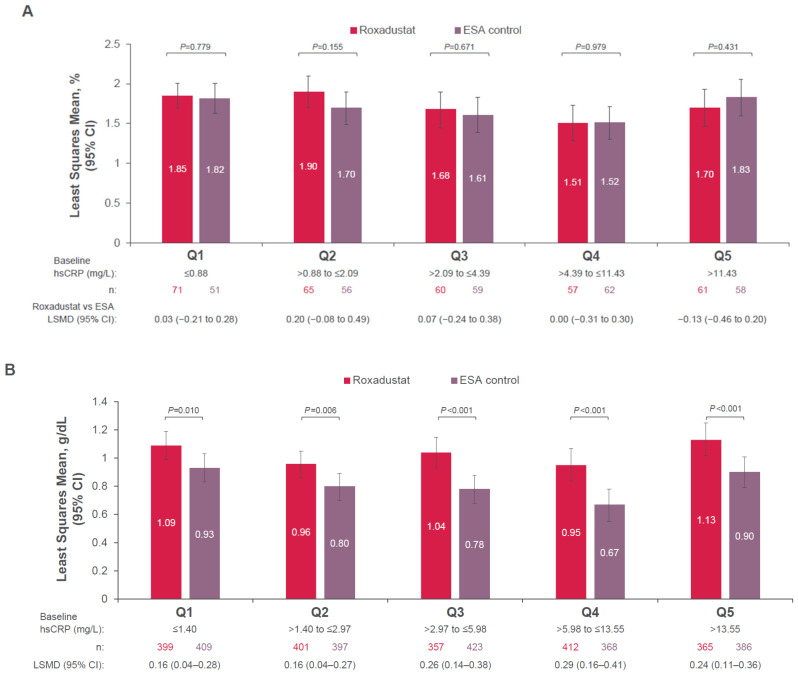
Mean Hemoglobin Levels Change From Baseline to Weeks 28 to 52 in the (**A**) NDD Population ^a,b^ and (**B**) DD Population ^c,d^. ANCOVA, analysis of covariance; CI, confidence interval; DD, dialysis-dependent; eGFR, estimated glomerular filtration rate; ESA, erythropoiesis-stimulating agent; Hb, hemoglobin; hsCRP, high-sensitivity C-reactive protein; LS, least squares; LSMD, least squares mean difference; MAR, missing at random; NDD, non-dialysis-dependent; Q, quintile. ^a^ Change in Hb from baseline to mean during Weeks 28 to 52 was analyzed using an ANCOVA model with the following fixed effects covariates at baseline: Hb and eGFR values in continuous scales and treatment group. ^b^ Monotone missing data were imputed by regression within each treatment group using Monte Carlo Markov Chain MAR-based multiple imputation. Adjusted LS means, their difference, and corresponding CIs were generated from datasets where missing data were imputed using MAR-based multiple imputation by treatment group, with baseline Hb and baseline eGFR as variables. ^c^ Change in Hb from baseline to mean during Weeks 28 to 52 was analyzed using an ANCOVA model with baseline Hb as a covariate and cardiovascular/cerebrovascular/thromboembolic history, geographical region, incident vs. stable dialysis (≤4 months vs. >4 months, respectively), and treatment groups as fixed effects. ^d^ Monotone missing data were imputed by regression within each treatment group using Monte Carlo Markov Chain MAR-based multiple imputation. Adjusted LS means, their difference and corresponding CIs were generated from datasets where missing data were imputed using MAR-based multiple imputation by treatment group, with baseline Hb, cardiovascular/cerebrovascular/thromboembolic history, geographical region, and incident vs. stable dialysis (≤4 months vs. >4 months, respectively) as predictor variables.

In patients with NDD CKD treated with roxadustat, the mean change from baseline to Weeks 28–52 in hemoglobin levels (LSM change: 1.72 g/dL; 95% CI, 1.64–1.80) was non-inferior to ESA (LSM change 1.70 g/dL; 95% CI, 1.61–1.79 with the 95% CI for the difference [−0.10, 0.14]). Non-inferiority was established in NDD patients regardless of baseline hsCRP since the lower limit of the 95% CI for the difference in each hsCRP quintile had a lower limit that exceeded −0.75 g/dL (Figure 2A).

For DD patients with known baseline hsCRP, the non-inferiority of roxadustat to ESA was demonstrated for each of the five hsCRP quintiles for inflammation status since the lower limit of the 95% CI for the difference exceeded −0.75 g/dL (Figure 2B). The mean change from baseline to Weeks 28–52 in hemoglobin levels was significantly greater in patients treated with roxadustat (LSM change, 1.03 g/dL; 95% CI, 0.98–1.08) compared with patients treated with ESA (LSM change, 0.81 g/dL; 95% CI, 0.77–0.86 with the 95% CI for the difference [0.17, 0.27]), regardless of baseline inflammation status (Figure 2B).

**Figure 3:**
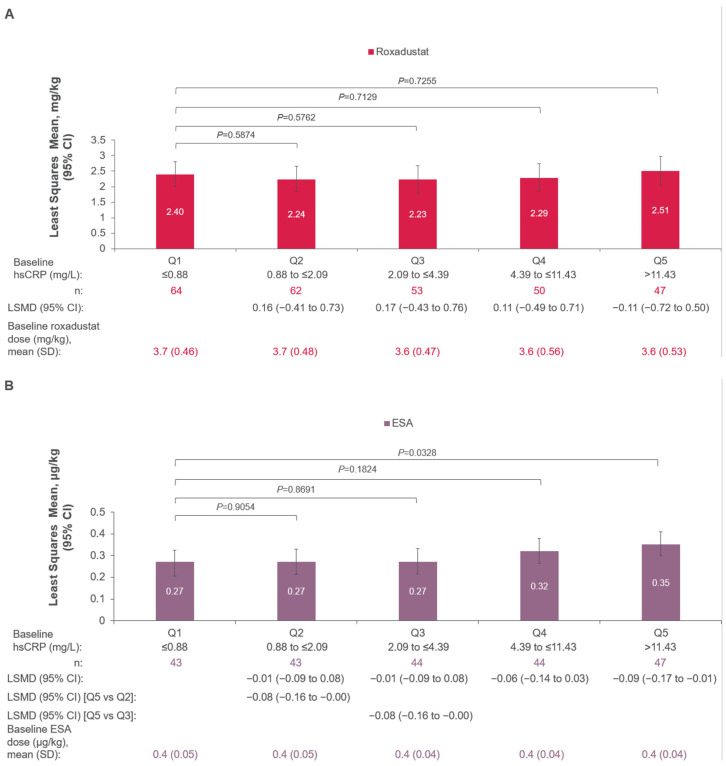
Mean weekly total (**A**) roxadustat dose ^a^ and (**B**) ESA dose ^a,b^ at week 24 in the NDD population. hsCRP quintile cut-off is based on subjects with non-missing baseline hsCRP values. Monotone missing data were imputed by regression within each treatment group using Monte Carlo Markov Chain MAR-based multiple imputation. ANCOVA, analysis of covariance; CI, confidence interval; eGFR, estimated glomerular filtration rate; ESA, erythropoiesis-stimulating agent; Hb, hemoglobin; hsCRP, high-sensitivity C-reactive protein; LSMD, least squares mean difference; MAR, missing at random; NDD, non-dialysis-dependent; Q, quintile. ^a^ Quintile comparison was made using an ANCOVA model with baseline Hb and baseline eGFR as covariates, and quintile rank, treatment group, and history of cardiovascular/cerebrovascular/thromboembolic disease (yes vs. no) as fixed effects. ^b^ The ESA used was darbepoetin alfa.

After 24 weeks of treatment, the roxadustat dose did not increase in patients with NDD CKD across hsCRP quintiles (LSM of hsCRP Q1–5: 2.40, 2.24, 2.23, 2.29, and 2.51 mg/kg, respectively; Figure 3A). The ESA dose was higher at Week 24 for patients with the highest hsCRP levels (e.g., Q5; >11.43 mg/L) compared with those with the lowest hsCRP levels (e.g., Q1; ≤0.88 mg/L; LSMD [Q1–Q5], −0.09; 95% CI, −0.17 to −0.01; *p* = 0.0328; Figure 3B). Patients with the highest baseline hsCRP had a higher LSM ESA dose compared with those in hsCRP quintile 2 (LSMD [Q2–Q5], −0.08; 95% CI, −0.16 to −0.004; *p* = 0.0405) and hsCRP quintile 3 (LSMD [Q3–Q5], −0.08; 95% CI, −0.16 to −0.001; *p* = 0.0462; Figure 3B).

**Figure 4:**
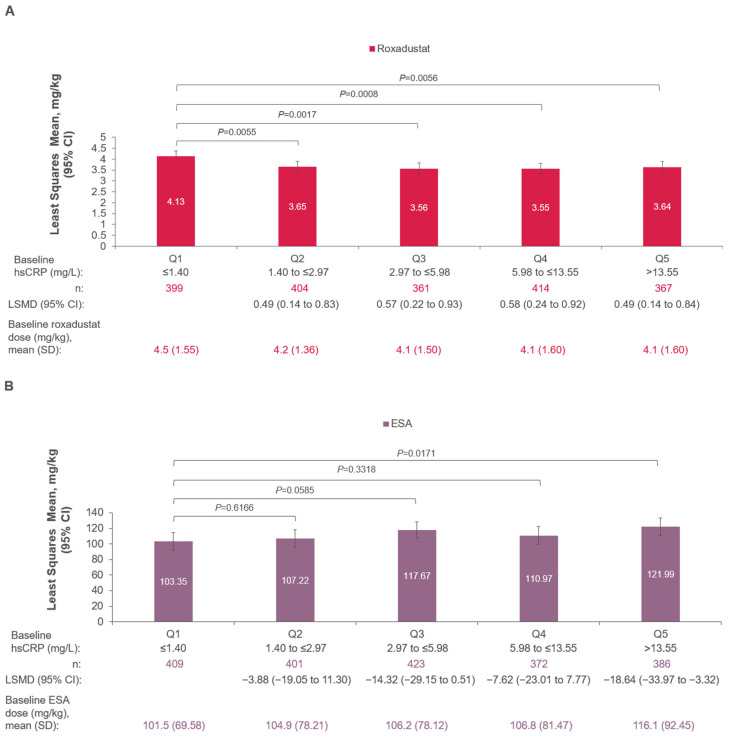
Mean weekly total (**A**) roxadustat dose ^a^ and (**B**) ESA dose ^a,b^ at week 24 in the DD population. hsCRP quintile cut-off is based on subjects with non-missing baseline hsCRP values. Monotone missing data were imputed by regression within each treatment group using Monte Carlo Markov Chain MAR-based multiple imputation. ANCOVA, analysis of covariance; CI, confidence interval; DD, dialysis-dependent; eGFR, estimated glomerular filtration rate; ESA, erythropoiesis-stimulating agent; Hb, hemoglobin; hsCRP, high-sensitivity C-reactive protein; LSMD, least squares mean difference; MAR, missing at random; Q, quintile; US, United States. ^a^ Quintile comparison was made using an ANCOVA model with baseline Hb as a covariate and study, quintile rank, study-by-quintile-rank interaction, region (US, Europe, Other), treatment group, and history of cardiovascular/cerebrovascular/thromboembolic disease (yes vs. no) as fixed effects. ^b^ Patients treated with darbepoetin alfa (µg/kg) were pooled with patients treated with epoetin alfa (IU/kg); units were converted to mg/kg using this formula: IU/kg × 0.67 = mg/kg.

In patients with DD CKD, roxadustat doses at Week 24 were significantly lower in patients with higher baseline hsCRP levels compared with the lowest baseline hsCRP level (LSM of hsCRP Q1–5: 4.13, 3.65, 3.56, 3.55, and 3.64 mg/kg, respectively; Figure 4A). In patients with DD CKD, the ESA dose was higher at Week 24 for patients with higher baseline hsCRP levels (Q5; >13.55 mg/L) compared with lower levels, e.g., quintile 1 (≤1.40 mg/L; LSMD [Q1–Q5], −18.64; 95% CI, −33.97 to −3.32; *p* = 0.0171; Figure 4B).

### 3.3. Iron Parameters

For patients with NDD CKD, ferritin and serum iron changes from baseline to Weeks 28–52 were not different between the roxadustat and ESA treatment groups (Table 1). There was a decrease in TSAT levels with roxadustat treatment compared with ESA treatment (LSMD −3.02%; 95% CI, −4.45 to −1.58; *p* < 0.001) for the combined group. TSAT levels in the roxadustat group were lower compared with those in the ESA group for patients in quintile 1 (≤88 mg/L; LSMD, −3.61; 95% CI, −7.01 to −0.20; *p* = 0.038), quintile 4 (>4.39 to ≤11.43 mg/L; LSMD, −3.86%; 95% CI, −6.83 to −0.89; *p* = 0.011), and quintile 5 (>11.43 mg/L; LSMD, −4.18%; 95% CI, −7.94 to −0.42; *p* = 0.029; Figure S1A).

For patients with DD CKD, reductions from baseline in ferritin levels at Weeks 28–52 and hepcidin levels at Week 24 were significant for patients treated with roxadustat compared with ESA regardless of baseline inflammation levels. Serum iron levels were significantly increased in the roxadustat group compared with the ESA group, regardless of baseline hsCRP levels. The change from baseline in TSAT levels was not significantly different between roxadustat- and ESA-treated patients across hsCRP quintiles (Table 2; Figure S1B).

### 3.4. Safety

For patients with NDD CKD, the overall incidence of TEAEs was similar with roxadustat (91.6%) and ESA (92.5%; Table S4). The overall incidences of TEAEs across hsCRP quintiles in the NDD CKD population treated with roxadustat (IR/100 Y) were 266.4, 193.1, 245.9, 383.3, and 319.5, for Q1–Q5, respectively, and for ESA, they were 176.7, 240.1, 212.2, 223.3, and 276.7 for Q1–Q5, respectively (Table 3). Among patients with NDD CKD, the most common TEAEs (with an incidence of ≥10%) were hypertension, end-stage kidney failure, decrease in eGFR, and hyperkalemia. The percentages and IRs of TEAEs were generally comparable between roxadustat and ESA across hsCRP quintiles (Table 4).

For patients with DD CKD, the overall incidence of TEAEs was similar for roxadustat (86.5%) and ESA (85.7%; Table S4). The overall incidence of TEAEs was numerically greater for patients with DD CKD treated with roxadustat in the highest hsCRP quintiles (IR/100 PEY, Q1–5: 47.8, 48.3, 52.1, 53.9, and 57.7, respectively) compared with ESA (Q1–5: 42.5, 43.0, 46.5, 45.2, and 49.1, respectively; Table 5). Among patients with DD CKD, the most common TEAEs were hypertension, diarrhea, pneumonia, arteriovenous fistula thrombosis, and headache; the percentages and IRs of TEAEs were generally comparable between roxadustat and ESA across hsCRP quintiles (Table 6). The percentages of patients with arteriovenous fistula thromboses were generally similar for the roxadustat and ESA groups overall; in patients with baseline hsCRP > 13.55 mg/L (Q5), the percentage of patients with arteriovenous fistula thrombosis was higher in the roxadustat-treated group (12.5%; IR/100 PEY, 8.0) compared with the ESA-treated group (7.3%; IR/100 PEY, 4.0). Infections did not occur in ≥10% of this patient population in either treatment group (Table 6).

## 4. Discussion

The present post hoc exploratory analysis evaluated two different patient populations treated with roxadustat from the ALPINE program. The NDD CKD population had lower hsCRP levels at baseline, with more patients in both treatment arms meeting the criteria for being iron-deplete compared with the DD CKD population; this difference in iron status at baseline in the two populations may be a result of more frequent iron supplementation [22].

Independent of baseline hsCRP level, roxadustat increased hemoglobin levels in a mnner comparable to ESA, with a similar safety profile, in patients with NDD CKD. In the NDD patient population, roxadustat doses did not increase after 24 weeks of treatment in patients with higher hsCRP levels at baseline. Conversely, patients with NDD CKD with high baseline hsCRP levels required higher ESA doses at Week 24 compared with patients with lower hsCRP levels at baseline. Roxadustat also increased hemoglobin levels in a manner comparable to ESA, with a similar safety profile, in patients with DD CKD independent of baseline inflammation status. Patients with the highest baseline hsCRP levels had higher incidences of diarrhea, nausea, and arteriovenous fistula thrombosis when treated with roxadustat compared with those treated with ESA. Likewise in the DD population, roxadustat doses did not increase at Week 24 in patients with higher hsCRP levels at baseline. Patients with DD CKD who had higher baseline hsCRP levels required higher ESA doses after 24 weeks of treatment compared with patients with lower baseline hsCRP levels. The percentages of TEAEs were generally similar between patients treated with roxadustat or ESA, regardless of baseline hsCRP levels.

Inflammation has been reported in approximately 30–50% of patients with CKD [23]. Previous studies have determined that patients with inflammation may not respond adequately to ESAs [7,8]. Three prior studies conducted in Japan with smaller patient populations than the present post hoc pooled analysis have also found that roxadustat was effective, without increasing the dose, regardless of baseline inflammation status [24,25,26]. One of these studies included patients receiving hemodialysis who were either ESA-naive (n = 75) or switched to roxadustat from ESAs (n = 164); a subgroup analysis of this study reported that, among patients with hsCRP levels ≥28.57 nmol/L compared with those who had hsCRP levels <28.57 nmol/L, the mean changes in hemoglobin levels from baseline to the end of treatment were comparable for ESA-naive patients and greater for ESA-converted patients [24]. Similarly, in a study of patients with NDD CKD (N = 262), the dose of roxadustat was not affected by inflammation status, whereas patients with elevated hsCRP treated with darbepoetin alfa required higher doses to maintain target hemoglobin levels [25]. In another previous study, inflammation was found to potentially impact the dose of darbepoetin alfa, but not roxadustat, in patients with anemia of CKD receiving hemodialysis (N = 303) [26]. Taken together, the findings from these studies and the current post hoc analysis suggest that roxadustat is effective regardless of inflammation status. Compared with ESAs, roxadustat is superior in reducing hepcidin levels, thus improving iron bioavailability [22,27]. In contrast with ESAs, roxadustat stimulates the production of endogenous erythropoietin, thereby exposing patients to lower erythropoietin plasma levels compared with ESA treatment [4].

In the DD CKD population, patients treated with roxadustat had significant reductions in ferritin levels and significant increases in serum iron at Weeks 28–52 relative to ESA at all hsCRP levels. Upon erythropoiesis stimulation in patients with CKD, there is an abrupt decrease in ferritin levels as hemoglobin levels rise, thereby indicating iron store mobilization. ESAs stimulate erythropoiesis but decrease ferritin and serum iron levels, thus requiring iron supplementation to increase hemoglobin levels [28]. In contrast, roxadustat stimulates erythropoiesis, decreases ferritin levels, and increases serum iron levels [22]; in the present study, these changes occurred with roxadustat treatment, regardless of inflammation status.

In patients with NDD CKD, TSAT levels decreased slightly following roxadustat treatment and increased with ESA treatment. In patients with DD CKD, TSAT levels decreased at similar levels with both roxadustat and ESA treatment, and patients with lower levels of baseline hsCRP had greater declines in TSAT levels. In the DD population, roxadustat-treated patients had a significantly greater decrease in ferritin and increased serum iron relative to ESA-treated subjects. In a previous pooled analysis, patients with NDD CKD treated with roxadustat had a decrease in TSAT relative to baseline, whereas TSAT was unchanged in placebo recipients, and in patients with DD CKD, the reduction in TSAT levels was similar in the roxadustat and ESA treatment groups [29]. TSAT is derived from the ratio of serum iron to the total iron-binding capacity (TIBC). Therefore, if serum iron levels and TIBC increase concomitantly, TSAT levels may not change or may decrease even though iron mobilization and utilization are improved relative to baseline. Consequently, TSAT may not be an ideal iron parameter for assessing the impact of roxadustat treatment on iron stores and their mobilization. In a previous analysis, there were greater increases in TIBC levels in patients with NDD or DD CKD receiving roxadustat compared with patients receiving placebo or ESA [29]. Individual assessments of serum iron levels and TIBC may be considered as markers of iron availability in patients treated with roxadustat, especially for patients with target hemoglobin levels. Additionally, oral or intravenous iron supplementation generally increases TSAT and ferritin levels. In the DOLOMITES study, a greater amount of intravenous iron was administered to ESA patients [17]. This may confound some of the iron parameter results observed in the overall NDD population.

Regardless of baseline inflammation levels, the percentages of TEAEs were generally similar between roxadustat and ESA. Previous pooled analyses that included patients with NDD CKD [30] and patients with DD CKD [31] found no increased risk of all-cause mortality with roxadustat compared with ESA; this is consistent with the current analysis, as overall mortality across all treatment groups was comparable, as was expected in these patient populations. Thromboembolism, hyperkalemia, and seizure are TEAEs that should be monitored in patients treated with roxadustat and ESAs; generally, TEAEs do not appear to affect the discontinuation of roxadustat more than ESA comparators [30,31].

The incidence of arteriovenous fistula thrombosis was higher with roxadustat treatment compared with ESA treatment in patients with DD CKD. A large meta-analysis consisting of more than 24,000 patients did not find a significant difference in the occurrence of arteriovenous fistula thrombosis in patients treated with HIF-PHIs compared with ESAs [32]. Access thrombosis may lead to inpatient admission or missed dialysis sessions [33]. In patients with DD CKD, the rates of vascular access thrombosis in patients treated with roxadustat were highest in the first 12 weeks after treatment initiation, at hemoglobin levels >12 g/dL, and in the setting of hemoglobin rise >2 g/dL over 4 weeks. Hemoglobin levels should be monitored, and it is recommended to adjust the dose of roxadustat using the dose adjustment rules per the label to avoid hemoglobin levels >12 g/dL and a rise in hemoglobin levels >2 g/dL over 4 weeks [34]. The number of patients with hyperkalemia was similar in roxadustat and ESA recipients with DD CKD across all hsCRP quintiles. A greater number of TEAEs leading to discontinuation occurred in patients with DD CKD who received roxadustat compared with patients who received ESAs [31]. In both the NDD and DD populations, the incidence of pneumonia was greater in patients with high baseline hsCRP levels compared to those with low baseline hsCRP levels in both treatment arms. This post hoc analysis was not powered to test for significant differences in TEAEs in patients treated with roxadustat or ESA; these results should be cautiously interpreted as hypothesis-generating.

Several professional societies worldwide have released guidelines or recommendations for the use of HIF-PHIs for the management of anemia of CKD [35,36,37,38]. These guidelines recommend the use of HIF-PHIs for the treatment of anemia of CKD, including in patients with ESA hyporesponsiveness [35,36,37,38]. The European Renal Association guidelines recommend that the use of HIF-PHIs be considered in patients with NDD CKD or patients with DD CKD receiving PD when the patient prefers an oral treatment (for such reasons as accessibility, convenience, ease of administration, and no storage requirements), there are challenges to beginning or receiving ESAs (e.g., phobia of needles or inability to self-administer ESAs), there are challenges to the administration of iron therapy or increased iron availability is desired, the patient experiences ESA hyporesponsiveness or intolerance, and there is a chronic inflammatory state (CRP levels ≥3 mg/L). HIF-PHIs can be considered in patients with DD CKD receiving HD when the patient prefers an oral treatment, the patient is receiving HD at home, there is a hypersensitivity/unavailability of intravenous iron, the patient experiences ESA hyporesponsiveness or intolerance, and there is a chronic inflammatory state (CRP levels ≥3 mg/L) [36]. The potential benefits of HIF-PHIs, including roxadustat, in patients who are hyporesponsive to treatment with ESAs require further elucidation, and future research in this field should be inclusive of patients regardless of their inflammation status.

There were several strengths and limitations of this post hoc, pooled analysis. The studies included in these analyses constitute a global program with similar methodologies, including similar hemoglobin target levels, which limits the concerns of internal validity and strengthens the global generalization of these results to both the NDD CKD and DD CKD patient populations. One potential weakness in this analysis that could lead to a systematic departure of results (i.e., bias) is the differing DD nature of patients in these studies, which included stable dialysis and/or incident dialysis patients. A further investigation of the efficacy and safety results in more narrowly defined pools may be worthwhile, but we suggest that the strength of our analysis lies in the large available pool across patients with DD CKD. Doses may differ between the NDD and DD CKD patient populations based on residual renal function and the pathophysiology of each disease state. Patients with chronic inflammatory disease were excluded from these studies, which may have reduced the power of this analysis in detecting differences in patients with or without inflammation. For patients with DD CKD, the studies included in this analyses were open-label, which may lead to a potential bias against the new medication when reporting TEAEs and study drug discontinuation for patients treated with roxadustat compared with the standard of care (ESAs) [39]. The limited number of ESA-treated patients in the NDD patient population from the DOLOMITES study did not allow for sufficient power to assess cardiovascular events.

## 5. Conclusions

The results of this study suggest that roxadustat is a viable treatment for patients with anemia of CKD, with or without dialysis and irrespective of inflammation status. More stable dosing is to be expected of the roxadustat treatment compared to the tendency for dose increases with ESA at higher hsCRP levels observed in this pooled post hoc exploratory analysis. Further investigation in real-world studies of roxadustat in patients with inflammation, particularly those who may be hyporesponsive to ESAs, is required.

## Figures and Tables

**Table 1:** Change from baseline in iron parameters (ferritin, serum iron, and TSAT) to weeks 28‒52 in the NDD patient population.

	hsCRP Q1 ≤0.88 mg/L		hsCRP Q2 >0.88–≤2.09 mg/L		hsCRP Q3 >2.09–≤4.39 mg/L		hsCRP Q4 >4.39–≤11.43 mg/L		hsCRP Q5 >11.43 mg/L	
	Roxadustat n = 71	ESA n = 53	Roxadustat n = 66	ESA n = 55	Roxadustat n = 63	ESA n = 59	Roxadustat n = 59	ESA n = 64	Roxadustat n = 61	ESA n = 61
**Ferritin (µg/L)**										
n	69	51	65	55	59	57	55	61	59	56
Baseline, mean	229.65	184.67	200.36	194.73	203.24	213.77	274.44	243.50	270.54	268.82
Adjusted LS mean change (SE)	−20.54 (17.55)	−44.31 (19.73)	−52.95 (11.85)	−42.13 (12.76)	−33.30 (14.48)	−14.36 (15.31)	−60.37 (21.54)	−39.91 (20.76)	−53.11 (26.73)	−65.18 (26.48)
95% CI	−54.94, 13.85	−82.98, −5.64	−76.17, −29.73	−67.13, −17.12	−61.69, −4.92	−44.37, 15.64	−102.59, −18.16	−80.60, 0.79	−105.49, −0.72	−11.7.08, −13.29
LSMD (95% CI)	23.77 (−28.08, 75.63)		−10.83 (−44.98, 23.33)		−18.94 (−60.62, 22.74)		−20.47 (−79.52, 38.59)		12.07 (−62.01, 86.16)	
*p* value	0.369		0.534		0.373		0.497		0.749	
**Serum iron (µg/dL)**										
n	69	51	65	55	59	57	55	61	59	56
Baseline, mean	71.34	71.03	74.36	64.85	64.37	63.26	58.58	56.46	44.71	45.25
Adjusted LS mean change (SE)	6.49 (3.53)	13.13 (4.31)	4.27 (3.09)	−0.08 (3.22)	7.47 (2.85)	5.67 (2.97)	11.12 (3.37)	13.76 (3.14)	13.51 (3.38)	20.30 (3.30)
95% CI	−0.43, 13.41	4.68, 21.58	−1.78, 10.32	−6.39, 6.24	1.88, 13.06	−0.15, 11.49	4.51, 17.72	7.60, 19.91	6.88, 20.15	13.83, 26.78
LSMD (95% CI)	−6.65 (−17.51, 4.21)		4.34 (−4.52, 13.21)		1.80 (−6.30, 9.89)		−2.64 (−11.79, 6.52)		−6.79 (−16.17, 2.59)	
*p* value	0.230		0.337		0.663		0.572		0.156	
**TSAT (%)**										
n	69	51	65	55	59	57	55	61	59	56
Baseline, mean	26.91	27.29	27.57	25.25	24.00	24.19	22.95	21.85	18.14	18.13
Adjusted LS mean change (SE)	1.03 (1.13)	4.63 (1.33)	0.39 (1.12)	1.49 (1.18)	0.85 (1.15)	2.58 (1.17)	1.76 (1.08)	5.62 (1.02)	3.40 (1.36)	7.58 (1.33)
95% CI	−1.18, 3.24	2.03, 7.23	−1.81, 2.59	−0.82, 3.80	−1.41, 3.10	0.28, 4.88	−0.36, 3.88	3.61, 7.62	0.73, 6.07	4.97, 10.18
LSMD (95% CI)	−3.61 (−7.01, −0.20)		−1.10 (−4.30, 2.10)		−1.73 (−4.97, 1.51)		−3.86 (−6.83, −0.89)		−4.18 (−7.94, −0.42)	
*p* value	0.038		0.501		0.295		0.011		0.029	

CI, confidence interval; ESA, erythropoiesis-stimulating agent; hsCRP, high-sensitivity C-reactive protein; LS, least squares; LSMD, least squares mean difference; NA, not available; NDD, non-dialysis-dependent; Q, quintile; SE, standard error; TEAE, treatment-emergent adverse event; TSAT, transferrin saturation.

**Table 2:** Change from baseline in iron parameters to week 24 (hepcidin) and weeks 28‒52 (ferritin, serum iron, and TSAT) in the patients with DD CKD.

	hsCRP Q1 ≤1.4 mg/L		hsCRP Q2 >1.4–≤2.97 mg/L		hsCRP Q3 >2.97–≤5.98 mg/L		hsCRP Q4 >5.98–≤13.545 mg/L		hsCRP Q5 >13.545 mg/L	
	Roxadustat n = 405	ESA n = 413	Roxadustat n = 411	ESA n = 402	Roxadustat n = 373	ESA n = 440	Roxadustat n = 433	ESA n = 381	Roxadustat n = 400	ESA n = 414
**Ferritin (µg/L)**										
n	390	404	391	391	347	407	398	354	349	368
Baseline, mean	534.03	533.29	549.00	570.03	596.13	659.16	649.34	684.33	733.24	682.07
Adjusted LS mean change (SE)	−189.70 (16.07)	−98.38 (16.18)	−215.23 (17.16)	−130.62 (16.95)	−208.58 (19.90)	−163.01 (19.55)	−205.73 (20.49)	−142.76 (21.20)	−257.78 (27.04)	−177.08 (25.82)
95% CI	−221.19, −158.20	−130.10, −66.65	−248.86, −181.60	−163.86, −97.39	−247.60, −169.57	−201.34, −124.69	−245.90, −165.55	−184.32, −101.21	−310.80, −204.76	−227.69, −126.47
LSMD (95% CI)	−91.32 (−129.56, −53.08)		−84.60 (−125.16, −44.05)		−45.57 (−91.31, 0.18)		−62.97 (−108.56, −17.37)		−80.71 (−141.27, −20.14)	
*p* value	<0.001		<0.001		0.051		0.007		0.009	
**Serum iron (µg/dL)**										
n	390	404	391	391	347	407	398	354	349	368
Baseline, mean	83.86	81.62	73.74	71.65	69.46	71.30	63.79	66.48	58.74	57.07
Adjusted LS mean change (SE)	−3.74 (1.92)	−17.29 (1.74)	1.38 (1.83)	−9.11 (1.67)	2.74 (1.99)	−8.88 (1.78)	7.07 (1.87)	−2.23 (1.84)	7.60 (1.97)	−1.45 (1.78)
95% CI	−7.50, 0.02	−20.70, −13.88	−2.21, 4.97	−12.39, −5.83	−1.16, 6.65	−12.36, −5.41	3.40, 10.74	−5.84, 1.37	3.75, 11.46	−4.94, 2.03
LSMD (95% CI)	13.55 (9.28, 17.82)		10.49 (6.34, 14.64)		11.63 (7.35, 15.90)		9.31 (5.29, 13.33)		9.06 (4.88, 13.23)	
*p* value	<0.001		<0.001		<0.001		<0.001		<0.001	
**TSAT (%)**										
n	390	404	389	389	347	407	398	353	349	368
Baseline, mean	37.40	36.54	33.42	32.95	32.71	33.86	30.74	31.52	29.45	28.01
Adjusted LS mean change (SE)	−6.64 (0.71)	−7.45 (0.66)	−5.06 (0.71)	−4.39 (0.69)	−4.97 (0.71)	−5.60 (0.67)	−2.15 (0.77)	−2.80 (0.75)	−2.29 (0.78)	−2.68 (0.71)
95% CI	−8.03, −5.24	−8.73, −6.16	−6.46, −3.66	−5.74, −3.05	−6.36, −3.58	−6.91, −4.29	−3.65, −0.64	−4.27, −1.33	−3.82, −0.77	−4.08, −1.29
LSMD (95% CI)	0.81 (−0.75, 2.37)		−0.67 (−2.30, 0.96)		0.63 (−0.96, 2.22)		0.65 (−0.99, 2.30)		0.39 (−1.32, 2.10)	
*p* value	0.307		0.422		0.440		0.438		0.653	
**Hepcidin (ng/mL)**										
n	375	386	391	382	346	412	393	355	347	363
Baseline, mean	186.21	185.89	187.17	181.32	200.31	204.27	196.84	212.14	258.77	238.15
Adjusted LS mean change (SE)	−47.02 (6.15)	−23.31 (6.20)	−42.50 (5.29)	−23.09 (5.54)	−52.72 (6.91)	−32.80 (6.49)	−43.29 (6.94)	−22.46 (7.11)	−90.25 (7.64)	−72.34 (7.00)
95% CI	−59.07, −34.97	−35.46, −11.17	−52,87, −32.13	−33.96, −12.23	−66.15, −39.08	−45.53, −20.08	−56.90, −29.68	−36.39, −8.53	−105.23, −75.28	−86.05, −58.62
LSMD (95% CI)	−23.71 (−38.90, −8.51)		−19.40 (−32.77, −6.04)		−19.81 (−35.49, −4.13)		−20.83 (−37.08, −4.59)		−17.91 (−35.12, −0.71)	
*p* value	0.002		0.004		0.013		0.012		0.041	

CI, confidence interval; DD CKD, dialysis-dependent chronic kidney disease; ESA, erythropoiesis-stimulating agent; hsCRP, high-sensitivity C-reactive protein; LS, least squares; LSMD, least squares mean difference; NDD, non-dialysis-dependent; Q, quintile; SE, standard error; TEAE, treatment-emergent adverse event; TSAT, transferrin saturation.

**Table 3:** Summary of TEAEs in the NDD population by hsCRP quintile.

	hsCRP Q1 ≤0.88 mg/L		hsCRP Q2 >0.88–≤2.09 mg/L		hsCRP Q3 >2.09–≤4.39 mg/L		hsCRP Q4 >4.39–≤11.43 mg/L		hsCRP Q5 >11.43 mg/L	
	Roxadustat n = 71	ESA n = 53	Roxadustat n = 66	ESA n = 56	Roxadustat n = 63	ESA n = 59	Roxadustat n = 59	ESA n = 64	Roxadustat n = 61	ESA n = 61
**TEAE**										
n (%)	67 (94.4)	46 (86.6)	59 (89.4)	54 (96.4)	56 (88.9)	53 (89.8)	57 (96.6)	61 (95.3)	56 (91.8)	57 (93.4)
PY ^a^; FAIR	25.2; 266.4	26.0; 176.7	30.6; 193.1	22.5; 240.1	22.8; 245.9	25.0; 212.2	14.9; 383.3	27.3; 223.3	17.5; 319.5	20.6; 276.7
**Serious TEAE**										
n (%)	51 (71.8)	26 (49.1)	35 (53.0)	37 (66.1)	34 (54.0)	30 (50.8)	44 (74.6)	45 (70.3)	44 (72.1)	43 (70.5)
PY ^a^; FAIR	80.1; 63.7	69.1; 37.6	81.4; 43.0	65.9; 56.1	77.4; 43.9	75.3; 39.8	51.6; 85.3	70.4; 63.9	48.3; 91.0	63.1; 68.2
**TEAE leading to discontinuation of study drug ^b^**										
n (%)	8 (11.3)	0 (0)	5 (7.6)	2 (3.6)	3 (4.8)	1 (1.7)	4 (6.8)	6 (9.4)	5 (8.2)	2 (3.3)
PY ^a^; FAIR	121.8; 6.6	92.8; 0.0	115.3; 4.3	104.2; 1.9	104.4; 2.9	99.8; 1.0	101.4; 3.9	109.0; 5.5	90.9; 5.5	99.1; 2.0
**Grade ≥3 TEAE**										
n (%)	42 (59.2)	23 (43.4)	32 (48.5)	32 (57.1)	29 (46.0)	29 (49.2)	39 (66.1)	43 (67.2)	38 (62.3)	37 (60.7)
PY ^a^; FAIR	88.7; 47.4	74.6; 30.8	88.1; 36.3	71.0; 45.1	85.4; 34.0	74.5; 38.9	58.7; 66.4	73.4; 58.6	59.1; 64.3	71.2; 52.0
**TEAE leading to death**										
n (%)	4 (5.6)	4 (7.5)	3 (4.5)	1 (1.8)	4 (6.3)	4 (6.8)	8 (13.6)	11 (17.2)	11 (18.0)	11 (18.0)
PY ^a^; FAIR	122.6; 3.3	92.8; 4.3	117.6; 2.6	104.4; 1.0	104.6; 3.8	99.9; 4.0	101.6; 7.9	109.9; 10.0	91.6; 12.0	99.4; 11.1

ESA, erythropoiesis-stimulating agent; FAIR, follow-up adjusted incidence rate; hsCRP, high-sensitivity C-reactive protein; NDD, non-dialysis-dependent; PY, patient years; Q, quintile; TEAE, treatment-emergent adverse event. ^a^ PY for each patient = (first event occurrence or censor date − first dose date + 1)/365.25; incidence rate/100 PY = 100 × number of patients with events/PY. ^b^ “Drug Withdrawn” is checked for action taken with study treatment or “Discontinue Study” is checked as other action for these adverse events.

**Table 4:** TEAEs occurring in ≥10% of the NDD population by hsCRP quintile.

	hsCRP Q1 ≤0.88 mg/L		hsCRP Q2 >0.88–≤2.09 mg/L		hsCRP Q3 >2.09–≤4.39 mg/L		hsCRP Q4 >4.39–≤11.43 mg/L		hsCRP Q5 >11.43 mg/L	
TEAE, n (%) PY ^a^; FAIR	Roxadustat n = 71	ESA n = 53	Roxadustat n = 66	ESA n = 56	Roxadustat n = 63	ESA n = 59	Roxadustat n = 59	ESA n = 64	Roxadustat n = 61	ESA n = 61
Overall TEAEs	67 (94.4) 25.2; 266.4	46 (86.8) 26.0; 176.7	59 (89.4) 30.6; 193.1	54 (96.4) 22.5; 240.1	56 (88.9) 22.8; 245.9	53 (89.8) 25.0; 212.2	57 (96.6) 14.9; 383.3	61 (95.3) 27.3; 223.3	56 (91.8) 17.5; 319.5	57 (93.4) 20.6; 276.7
Anemia	3 (4.2) 120.5; 2.5	3 (5.7) 89.0; 3.4	3 (4.5) 114.5; 2.6	4 (7.1) 101.9; 3.9	2 (3.2) 103.8; 1.9	2 (3.4) 98.0; 2.0	2 (3.4) 101.4; 2.0	3 (4.7) 108.8; 2.8	4 (6.6) 88.5; 4.5	7 (11.5) 95.3; 7.3
Cardiac failure	4 (5.6) 118.1; 3.4	2 (3.8) 92.4; 2.2	1 (1.5) 116.9; 0.9	4 (7.1) 101.9; 3.9	5 (7.9) 101.1; 5.0	3 (5.1) 96.7; 3.1	6 (10.2) 96.2; 6.2	1 (1.6) 109.7; 0.9	2 (3.3) 90.5; 2.2	8 (13.1) 96.1; 8.3
Constipation	4 (5.6) 118.1; 3.4	3 (5.7) 88.9; 3.4	3 (4.5) 113.8; 2.6	3 (5.4) 102.6; 2.9	3 (4.8) 101.7; 2.9	4 (6.8) 95.0; 4.2	8 (13.6) 93.5; 8.6	1 (1.6) 108.0; 0.9	3 (4.9) 89.8; 3.3	4 (6.6) 93.7; 4.3
Diarrhea	4 (5.6) 117.0; 3.4	5 (9.4) 86.3; 5.8	4 (6.1) 110.7; 3.6	2 (3.6) 102.8; 1.9	6 (9.5) 94.3; 6.4	10 (16.9) 90.4; 11.1	6 (10.2) 95.3; 6.3	5 (7.8) 106.0; 4.7	8 (13.1) 86.0; 9.3	8 (13.1) 90.4; 8.8
Nausea	7 (9.9) 114.6; 6.1	2 (3.8) 91.3; 2.2	6 (9.1) 111.9; 5.4	7 (12.5) 98.6; 7.1	4 (6.3) 99.5; 4.0	8 (13.6) 91.2; 8.8	9 (15.3) 92.9; 9.7	5 (7.8) 104.9; 4.8	9 (14.8) 85.9; 10.5	3 (4.9) 95.9; 3.1
Vomiting	0 (0) 122.6; 0.0	1 (1.9) 91.9; 1.1	2 (3.0) 116.8; 1.7	6 (10.7) 97.5; 6.2	3 (4.8) 99.8; 3.0	7 (11.9) 91.9; 7.6	8 (13.6) 93.4; 8.6	5 (7.8) 105.5; 4.7	8 (13.1) 83.8; 9.5	0 (0) 99.4; 0.0
Asthenia	2 (2.8) 120.8; 1.7	1 (1.9) 91.0; 1.1	2 (3.0) 115.8; 1.7	0 (0) 104.4; 0.0	4 (6.3) 100.5; 4.0	2 (3.4) 97.2; 2.1	2 (3.4) 98.1; 2.0	7 (10.9) 101.7; 6.9	1 (1.6) 89.8; 1.1	1 (1.6) 98.1; 1.0
Edema, peripheral	13 (18.3) 107.4; 12.1	5 (9.4) 86.7; 5.8	10 (15.2) 104.9; 9.5	8 (14.3) 95.0; 8.4	5 (7.9) 99.4; 5.0	7 (11.9) 91.6; 7.6	9 (15.3) 90.0; 10.0	7 (10.9) 101.0; 6.9	12 (19.7) 78.7; 15.2	9 (14.8) 85.7; 10.5
Bronchitis	5 (7.0) 118.7; 4.2	4 (7.5) 88.2; 4.5	2 (3.0) 114.1; 1.8	3 (5.4) 101.8; 2.9	5 (7.9) 97.6; 5.1	3 (5.1) 96.8; 3.1	6 (10.2) 94.6; 6.3	2 (3.1) 108.2; 1.8	4 (6.6) 85.6; 4.7	6 (9.8) 92.0; 6.5
Pneumonia	4 (5.6) 119.4; 3.3	3 (5.7) 90.7; 3.3	3 (4.5) 115.8; 2.6	5 (8.9) 101.5; 4.9	4 (6.3) 103.6; 3.9	2 (3.4) 98.9; 2.0	5 (8.5) 99.6; 5.0	5 (7.8) 108.2; 4.6	9 (14.8) 84.5; 10.6	7 (11.5) 95.1; 7.4
Urinary tract infection	3 (4.2) 120.4; 2.5	2 (3.8) 91.7; 2.2	4 (6.1) 114.5; 3.5	3 (5.4) 102.1; 2.9	6 (9.5) 99.4; 6.0	6 (10.2) 95.2; 6.3	4 (6.8) 99.6; 4.0	4 (6.3) 107.5; 3.7	4 (6.6) 88.6; 4.5	12 (19.7) 87.3; 13.7
Viral upper respiratory tract infection	7 (9.9) 116.8; 6.0	3 (5.7) 90.6; 3.3	5 (7.6) 112.4; 4.4	6 (10.7) 96.2; 6.2	3 (4.8) 100.3; 3.0	6 (10.2) 88.8; 6.8	8 (13.6) 91.4; 8.7	6 (9.4) 104.3; 5.8	6 (9.8) 84.5; 7.1	4 (6.6) 94.2; 4.2
Arteriovenous fistula thrombosis	3 (4.2) 121.2; 2.5	2 (3.8) 91.6; 2.2	2 (3.0) 115.7; 1.7	3 (5.4) 101.1; 3.0	2 (3.2) 102.4; 2.0	0 (0) 99.9; 0.0	8 (13.6) 95.1; 8.4	3 (4.7) 108.2; 2.8	1 (1.6) 91.3; 1.1	2 (3.3) 98.1; 2.0
Fall	3 (4.2) 118.3; 2.5	0 (0) 92.8; 0.0	0 (0) 117.6; 0.0	3 (5.4) 102.1; 2.9	3 (4.8) 103.1; 2.9	2 (3.4) 97.4; 2.1	7 (11.9) 98.9; 7.1	0 (0) 109.9; 0.0	2 (3.3) 88.1; 2.3	4 (6.6) 94.9; 4.2
Glomerular filtration rate decrease	17 (23.9) 105.5; 16.1	7 (13.2) 86.2; 8.1	13 (19.7) 103.6; 12.6	14 (25.0) 86.5; 16.2	8 (12.7) 95.5; 8.4	9 (15.3) 88.9; 10.1	9 (15.3) 91.7; 9.8	11 (17.2) 99.0; 11.1	7 (11.5) 83.6; 8.4	8 (13.1) 95.0; 8.4
Hyperkalemia	9 (12.7) 108.7; 8.3	5 (9.4) 88.4; 5.7	8 (12.1) 107.8; 7.4	11 (19.6) 92.5; 11.9	8 (12.7) 94.6; 8.5	12 (20.3) 90.7; 13.2	9 (15.3) 91.7; 9.8	6 (9.4) 102.7; 5.8	4 (6.6) 88.0; 4.5	8 (13.1) 91.0; 8.8
Hyperphosphatemia	5 (7.0) 118.8; 4.2	4 (7.5) 87.6; 4.6	7 (10.6) 110.4; 6.3	3 (5.4) 100.3; 3.0	6 (9.5) 96.7; 6.2	1 (1.7) 99.4; 1.0	4 (6.8) 98.1; 4.1	4 (6.3) 106.2; 3.8	6 (9.8) 84.5; 7.1	3 (4.9) 97.0; 3.1
Iron deficiency	6 (8.5) 115.0; 5.2	2 (3.8) 89.3; 2.2	2 (3.0) 115.0; 1.7	3 (5.4) 101.6; 3.0	3 (4.8) 100.7; 3.0	3 (5.1) 98.2; 3.1	8 (13.6) 96.7; 8.3	8 (12.5) 103.6; 7.7	2 (3.3) 89.4; 2.2	9 (14.8) 83.4; 10.8
Back Pain	2 (2.8) 121.0; 1.7	1 (1.9) 92.3; 1.1	6 (9.1) 111.5; 5.4	3 (5.4) 102.2; 2.9	3 (4.8) 99.6; 3.0	1 (1.7) 98.8; 1.0	4 (6.8) 97.7; 4.1	7 (10.9) 103.0; 6.8	5 (8.2) 86.1; 5.8	5 (8.2) 93.8; 5.3
End-stage kidney failure	25 (35.2) 101.3; 24.7	15 (28.3) 80.0; 18.8	21 (31.8) 97.7; 21.5	24 (42.9) 81.3; 29.5	12 (19.0) 93.3; 12.9	18 (30.5) 85.3; 21.1	29 (49.2) 72.7; 39.9	27 (42.2) 89.4; 30.2	20 (32.8) 72.4; 27.6	22 (36.1) 80.7; 27.3
Dyspnea	8 (11.3) 115.3; 6.9	0 (0) 92.8; 0.0	3 (4.5) 114.9; 2.6	2 (3.6) 103.6; 1.9	3 (4.8) 102.2; 2.9	1 (1.7) 97.8; 1.0	2 (3.4) 100.5; 2.0	5 (7.8) 105.7; 4.7	8 (13.1) 83.9; 9.5	4 (6.6) 94.9; 4.2
Pruritus	4 (5.6) 118.5; 3.4	1 (1.9) 91.9; 1.1	2 (3.0) 115.7; 1.7	3 (5.4) 101.7; 3.0	1 (1.6) 102.7; 1.0	3 (5.1) 98.2; 3.1	8 (13.6) 92.4; 8.7	4 (6.3) 105.3; 3.8	5 (8.2) 84.8; 5.9	2 (3.3) 98.0; 2.0
Hypertension	22 (31.0) 99.4; 22.1	23 (43.4) 61.2; 37.6	15 (22.7) 99.2; 15.1	24 (42.9) 67.8; 35.4	22 (34.9) 81.9; 26.9	18 (30.5) 74.0; 24.3	21 (35.6) 71.0; 29.6	15 (23.4) 90.5; 16.6	16 (26.2) 73.8; 21.7	19 (31.1) 76.4; 24.9

ESA, erythropoiesis-stimulating agent; FAIR, follow-up adjusted incidence rate; hsCRP, high-sensitivity C-reactive protein; NDD, non-dialysis-dependent; PY, patient years; Q, quintile; TEAE, treatment-emergent adverse event. ^a^ PY for each patient = (first event occurrence or censor date − first dose date + 1)/365.25; incidence rate/100 PY = 100 × number of patients with events/PY.

**Table 5:** Summary of TEAEs in the DD population by hsCRP quintile.

	hsCRP Q1 ≤1.40 mg/L		hsCRP Q2 >1.40–≤2.97 mg/L		hsCRP Q3 >2.97–≤5.98 mg/L		hsCRP Q4 >5.98–≤13.545 mg/L		hsCRP Q5 >13.545 mg/L	
	Roxadustat n = 405 PEY ^a^ = 733.9	ESA n = 413 PEY ^a^ = 812.2	Roxadustat n = 411 PEY ^a^ = 750.2	ESA n = 402 PEY ^a^ = 790.1	Roxadustat n = 373 PEY ^a^ = 619.8	ESA n = 438 PEY ^a^ = 855.3	Roxadustat n = 433 PEY ^a^ = 712.4	ESA n = 381 PEY ^a^ = 743.9	Roxadustat n = 400 PEY ^a^ = 624.2	ESA n = 412 PEY ^a^ = 741.6
**TEAE**										
n (%)	351 (86.7)	345 (83.5)	362 (88.1)	340 (84.6)	323 (86.6)	398 (90.9)	384 (88.7)	336 (88.2)	360 (90.0)	364 (88.3)
IR/100 PEY	47.8	42.5	48.3	43.0	52.1	46.5	53.9	45.2	57.7	49.1
**Serious TEAE**										
n (%)	205 (50.6)	184 (44.6)	214 (52.1)	190 (47.3)	191 (51.2)	239 (54.6)	246 (56.8)	229 (60.1)	259 (64.8)	255 (61.9)
IR/100 PEY	27.9	22.7	28.5	24.0	30.8	27.9	34.5	30.8	41.5	34.4
**TEAE leading to discontinuation of study drug ^b^**										
n (%)	35 (8.6)	20 (4.8)	42 (10.2)	26 (6.5)	40 (10.7)	37 (8.4)	52 (12.0)	40 (10.5)	64 (16.0)	42 (10.2)
IR/100 PEY	4.8	2.5	5.6	3.3	6.5	4.3	7.3	5.4	10.3	5.7
**Grade ≥3 TEAE**										
n (%)	152 (37.5)	128 (31.0)	168 (40.9)	151 (37.6)	152 (40.8)	187 (42.7)	211 (48.7)	190 (49.9)	225 (56.3)	208 (50.5)
IR/100 PEY	20.7	15.8	22.4	19.1	24.5	21.9	29.6	25.5	36.0	28.0
**TEAE leading to death**										
n (%)	34 (8.4)	31 (7.5)	54 (13.1)	46 (11.4)	60 (16.1)	69 (15.8)	72 (16.6)	67 (17.6)	94 (23.5)	84 (20.4)
IR/100 PEY	4.6	3.8	7.2	5.8	9.7	8.1	10.1	9.0	15.1	11.3

DD, dialysis-dependent; ESA, erythropoiesis-stimulating agent; hsCRP, high-sensitivity C-reactive protein; IR, incidence rate; PEY, patient years; Q, quintile; TEAE, treatment-emergent adverse event. ^a^ PEY for each patient = ([last dose date − first dose date] + 1)/365.25. Incidence rate/100 PEY = 100 × number of patients with events/PEY. ^b^ “Drug Withdrawn” is checked for action taken with study treatment or “Discontinue Study” is checked as other action for these adverse events.

**Table 6:** TEAEs occurring in ≥10% of the DD population by hsCRP quintile.

	hsCRP Q1 ≤1.40 mg/L		hsCRP Q2 >1.40–≤2.97 mg/L		hsCRP Q3 >2.97–≤5.98 mg/L		hsCRP Q4 >5.98–≤13.545 mg/L		hsCRP Q5 >13.545 mg/L	
TEAE, n (%), IR	Roxadustat n = 405 PEY ^a^ = 733.9	ESA n = 413 PEY ^a^ = 812.2	Roxadustat n = 411 PEY ^a^ = 750.2	ESA n = 402 PEY ^a^ = 790.1	Roxadustat n = 373 PEY ^a^ = 619.8	ESA n = 438 PEY ^a^ = 855.3	Roxadustat n = 433 PEY ^a^ = 712.4	ESA n = 381 PEY ^a^ = 743.9	Roxadustat n = 400 PEY ^a^ = 624.2	ESA n = 412 PEY ^a^ = 741.6
Overall TEAEs	351 (86.7), 47.8	345 (83.5), 42.5	362 (88.1), 48.3	340 (84.6), 43.0	323 (86.6), 52.1	398 (90.9), 46.5	384 (88.7), 53.9	336 (88.2), 45.2	360 (90.0), 57.7	364 (88.3), 49.1
Diarrhea	55 (13.6), 7.5	42 (10.2), 5.2	46 (11.2), 6.1	48 (11.9), 6.1	36 (9.7), 5.8	43 (9.8), 5.0	49 (11.3), 6.9	53 (13.9), 7.1	54 (13.5), 8.7	39 (9.5), 5.3
Nausea	25 (6.2), 3.4	41 (9.9), 5.0	31 (7.5), 4.1	22 (5.5), 2.8	28 (7.5), 4.5	25 (5.7), 2.9	41 (9.5), 5.8	28 (7.3), 3.8	46 (11.5), 7.4	31 (7.5), 4.2
Pneumonia	32 (7.9), 4.4	36 (8.7), 4.4	37 (9.0), 4.9	33 (8.2), 4.2	27 (7.2), 4.4	56 (12.8), 6.5	48 (11.1), 6.7	38 (10.0), 5.1	35 (8.8), 5.6	38 (9.2), 5.1
Arteriovenous fistula-site complication	28 (6.9), 3.8	27 (6.5), 3.3	36 (8.8), 4.8	28 (7.0), 3.5	27 (7.2), 4.4	33 (7.5), 3.9	32 (7.4), 4.5	41 (10.8), 5.5	34 (8.5), 5.4	33 (8.0), 4.5
Arteriovenous fistula thrombosis	33 (8.1), 4.5	26 (6.3), 3.2	43 (10.5), 5.7	33 (8.2), 4.2	38 (10.2), 6.1	42 (9.6), 4.9	43 (9.9), 6.0	34 (8.9), 4.6	50 (12.5), 8.0	30 (7.3), 4.0
Pain in extremity	22 (5.4), 3.0	16 (3.9), 2.0	22 (5.4), 2.9	26 (6.5), 3.3	21 (5.6), 3.4	17 (3.9), 2.0	21 (4.8), 2.9	40 (10.5), 5.4	21 (5.3), 3.4	27 (6.6), 3.6
Headache	39 (9.6), 5.3	30 (7.3), 3.7	50 (12.2), 6.7	32 (8.0), 4.1	25 (6.7), 4.0	37 (8.4), 4.3	42 (9.7), 5.9	27 (7.1), 3.6	34 (8.5), 5.4	32 (7.8), 4.3
Hypertension	72 (17.8), 9.8	58 (14.0), 7.1	61 (14.8), 8.1	72 (17.9), 9.1	52 (13.9), 8.4	70 (16.0), 8.2	73 (16.9), 10.2	44 (11.5), 5.9	51 (12.8), 8.2	48 (11.7), 6.5
Hypotension	27 (6.7), 3.7	32 (7.7), 3.9	44 (10.7), 5.9	23 (5.7), 2.9	29 (7.8), 4.7	32 (7.3), 3.7	43 (9.9), 6.0	38 (10.0), 5.1	40 (10.0), 6.4	32 (7.8), 4.3

DD, dialysis-dependent; ESA, erythropoiesis-stimulating agent; hsCRP, high-sensitivity C-reactive protein; IR, incidence rate; PEY, patient exposure years; Q, quintile; TEAE, treatment-emergent adverse event. ^a^ PEY for each patient = ([last dose date − first dose date] + 1)/365.25; incidence rate/100 PEY = 100 × number of patients with events/PEY.

## Data Availability

Researchers may request access to anonymized participant-level data, trial-level data and protocols from Astellas-sponsored clinical trials at www.clinicalstudydatarequest.com. For the Astellas criteria on data sharing, see https://clinicalstudydatarequest.com/Study-Sponsors/Study-Sponsors-Astellas.aspx, accessed on 19 December 2024.

## Supplementary Materials

The following supporting information can be downloaded at: https://www.mdpi.com/article/10.3390/jcm14020303/s1, Appendix SA, Figure S1: Mean TSAT change from baseline to weeks 28–52 in the (A) NDD patient population and the (B) DD patient population; Table S1: Baseline demographics and disease characteristics; Table S2: Baseline demographics and disease characteristics in the NDD population by hsCRP quintile; Table S3: Baseline demographics and disease characteristics in the DD population by hsCRP quintile; Table S4: Safety in the overall NDD and DD populations.

## Trial Registration Numbers

NCT02021318; NCT02174731; NCT02273726; NCT02052310; NCT02278341.
