# Supplementary material for: Efficacy and Safety of Roxadustat in Patients with CKD: Pooled Analysis by Baseline Inflammation Status
Source: J Clin Med. 2025 Jan 7;14(2):303. doi: 10.3390/jcm14020303 (PMC11765649; doi:10.3390/jcm14020303)
Supplement: Supplementary file 1 [file jcm-14-00303-s001.zip › jcm-3366253-supplementary.pdf]

Efficacy and Safety of Roxadustat in Patients With Non–Dialysis-Dependent or Dialysis-Dependent Chronic Kidney Disease by Baseline Inflammation Status

Gabriel Choukroun[1]; Frank Strutz[2]; Alexander Harkavyi[3]; Vicki Santos[4]; Alina Jiletcovici[4]; Lucia Del Vecchio[5]

[1]CHU Amiens Picardie and Jules Verne University, Amiens, France; [2]DKD Helios Klinik Wiesbaden, KfH und Nierenzentrum-Rheumatologie Wiesbaden, Germany; [3]Astellas Pharma Europe, Addlestone, Surrey UK; [4]Astellas Pharma, Inc., Northbrook, IL, USA; [5]Sant'Anna Hospital, ASST Lariana, Como, Italy

Supplementary Material

Table S1. Baseline demographics and disease characteristics

Table S2. Baseline demographics and disease characteristics in the NDD population by hsCRP quintile

Table S3. Baseline demographics and disease characteristics in the DD population by hsCRP quintile

Table S4. Safety in the overall NDD and DD populations

Figure S1. Mean TSAT change from baseline to weeks 28–52 in the (A) NDD patient population and the (B) DD patient population

Appendix SA
S1. Supplementary Methods
S1.1. Target Hemoglobin Levels

For patients with NDD CKD, the target hemoglobin levels were 10.0 to 12.0 g/dL. In NDD CKD patients, the hemoglobin threshold, prior to randomization, was ≤10.5 g/dL.
Target hemoglobin levels were 10.0 to 12.0 g/dL for all studies that included patients with DD CKD (ROCKIES, SIERRAS, HIMALAYAS, and PYRENEES). In patients with DD CKD, the hemoglobin threshold, prior to randomization, ranged across the four studies from 9.0 to 12.0 g/dL.

Table S1. Baseline demographics and disease characteristics

	NDD population		DD population	
	**Roxadustat n=320**	**ESA n=293**	**Roxadustat n=2022**	**ESA n=2050**
Age,[a] y, mean (SD)	66.9 (13.6)	65.7 (14.4)	56.0 (14.9)	56.6 (14.6)
Gender, male, n (%)	144 (45.0)	129 (44.0)	1174 (58.1)	1195 (58.3)
Race, n (%)				
White	303 (94.7)	281 (95.9)	1416 (70.0)	1424 (69.5)
Black or African American	8 (2.5)	2 (0.7)	298 (14.7)	310 (15.1)
Asian	9 (2.8)	10 (3.4)	206 (10.2)	217 (10.6)
Native Hawaiian or other Pacific Islander	0 (0)	0 (0)	4 (0.2)	4 (0.2)
American Indian or Alaska native	0 (0)	0 (0)	33 (1.6)	45 (2.2)
Other	0 (0)	0 (0)	65 (3.2)	50 (2.4)
Body mass index, kg/m^2, mean (SD)	27.9 (5.76)	28.7 (6.06)	27.5 (6.48)	27.6 (6.51)
Hb, g/dL, mean (SD)	9.55 (0.75)	9.55 (0.69)	9.80 (1.29)	9.83 (1.29)
eGFR, mL/min/1.73 m^2, mean (SD)	20.32 (11.51)	20.34 (10.73)	—	—
Ferritin, µg/L, mean (SD)	233.6 (231.31)	227.2 (210.77)	616.8 (457.18)	629.6 (486.08)
Albumin (g/L), mean (SD)	39.4 (3.91)	39.2 (3.71)	37.6 (3.94)	37.5 (3.97)
Cardiovascular disease history, yes, n (%)	150 (46.9)	142 (48.5)	871 (43.1)	901 (44.0)
Type of dialysis,[b] n (%)				
Hemodialysis	—	—	1834 (90.7)	1874 (91.4)
Peritoneal dialysis	—	—	186 (9.2)	176 (8.6)
Dialysis vintage, n (%)				
≤4 months	—	—	691 (34.2)	702 (34.2)
>4 months	—	—	1331 (65.8)	1348 (65.8)
Iron status,[c] n (%)				
TSAT ≥20% and ferritin ≥100 ng/mL	181 (56.6)	152 (51.9)	1759 (87.0)	1789 (87.3)
TSAT <20% or ferritin <100 ng/mL	139 (43.4)	141 (48.1)	261 (12.9)	257 (12.5)

Systolic blood pressure, mm Hg, mean (SD)	137.19 (15.24)	137.53 (14.84)	140.91 (17.77)	140.57 (17.78)
Diastolic blood pressure, mm Hg, mean (SD)	74.87 (9.98)	75.24 (10.44)	77.51 (10.44)	77.10 (10.52)

ANOVA, analysis of variance; DD, dialysis-dependent; eGFR, estimated glomerular filtration rate; ESA, erythropoiesis-stimulating agent; Hb, hemoglobin; NDD, non–dialysis-dependent; SD, standard deviation; TSAT, transferrin saturation.

[a]Age at study entry.

[b]Two patients were missing in the roxadustat treatment group.

[c]Two patients were missing in the roxadustat treatment group and four patients were missing in the ESA treatment group.

Table S2. Baseline demographics and disease characteristics in the NDD population by hsCRP quintile

	hsCRP Q1 ≤0.88 mg/L		hsCRP Q2 >0.88 – ≤2.09 mg/L		hsCRP Q3 >2.09 – ≤4.39 mg/L		hsCRP Q4 >4.39 – ≤11.43 mg/L		hsCRP Q5 >11.43 mg/L	
	Rox n=71	ESA n=53	Rox n=66	ESA n=56	Rox n=63	ESA n=59	Rox n=59	ESA n=64	Rox n=61	ESA n=61
Age,[a] y, mean (SD)	65.6 (14.1)	62.9 (16.0)	65.7 (16.1)	62.6 (15.9)	69.0 (11.7)	65.0 (13.4)	68.5 (12.5)	67.4 (13.2)	65.7 (12.9)	70.1 (12.7)
Gender, male, n (%)	35 (49.3)	19 (35.8)	29 (43.9)	25 (44.6)	25 (39.7)	22 (37.3)	24 (40.7)	33 (51.6)	31 (50.8)	30 (49.2)
Race, n (%)										
White	68 (95.8)	50 (94.3)	62 (93.9)	52 (92.9)	58 (92.1)	58 (98.3)	57 (96.6)	61 (95.3)	58 (95.1)	60 (98.4)
Black or African American	1 (1.4)	0 (0)	1 (1.5)	0 (0)	3 (4.8)	0 (0)	1 (1.7)	1 (1.6)	2 (3.3)	1 (1.6)
Asian	2 (2.8)	3 (5.7)	3 (4.5)	4 (7.1)	2 (3.2)	1 (1.7)	1 (1.7)	2 (3.1)	1 (1.6)	0 (0)
Native Hawaiian or other Pacific Islander	0 (0)	0 (0)	0 (0)	0 (0)	0 (0.0)	0 (0)	0 (0)	0 (0)	0 (0)	0 (0)
American Indian or Alaska native	0 (0)	0 (0)	0 (0)	0 (0)	0 (0)	0 (0)	0 (0)	0 (0)	0 (0)	0 (0)
Other	0 (0)	0 (0)	0 (0)	0 (0)	0 (0)	0 (0)	0 (0)	0 (0)	0 (0)	0 (0)
Body mass index, kg/m^2, mean (SD)	25.6 (5.22)	25.7 (4.68)	26.5 (4.83)	26.8 (4.42)	28.9 (5.63)	30.3 (6.26)	28.9 (5.34)	29.1 (6.23)	30.1 (6.61)	31.2 (6.54)
Hb, g/dL, mean (SD)	9.61 (0.83)	9.54 (0.69)	9.58 (0.68)	9.43 (0.68)	9.59 (0.63)	9.62 (0.70)	9.54 (0.71)	9.66 (0.66)	9.39 (0.87)	9.50 (0.71)
eGFR, mL/min/1.73 m^2, mean (SD)	20.74 (11.62)	20.95 (11.97)	19.31 (11.02)	18.81 (10.49)	22.84 (13.29)	20.19 (10.63)	16.66 (9.01)	21.63 (11.87)	21.85 (11.47)	20.01 (8.55)
Ferritin, µg/L, mean (SD)	229.9 (213.46)	199.3 (210.69)	199.7 (146.05)	194.7 (156.42)	208.8 (158.55)	216.2 (170.49)	271.1 (354.57)	239.0 (240.67)	263.9 (237.60)	279.6 (248.30)
Albumin, g/L, mean (SD)	41.0 (3.45)	39.3 (3.51)	39.6 (3.64)	40.1 (3.49)	39.5 (4.17)	39.6 (3.71)	38.6 (3.11)	39.1 (3.87)	37.7 (4.40)	37.9 (3.66)
Cardiovascular disease history, yes, n (%)	35 (49.3)	23 (43.4)	24 (36.4)	32 (57.1)	26 (41.3)	29 (49.2)	33 (55.9)	21 (32.8)	32 (52.5)	37 (60.7)
Diabetes,[b] n (%)	32 (45.1)	25 (47.2)	23 (34.8)	19 (33.9)	35 (55.6)	29 (49.2)	27 (45.8)	30 (46.9)	30 (49.2)	34 (55.7)
Iron status, n (%)										
TSAT ≥20% or ferritin ≥100 ng/mL	51 (71.8)	34 (64.2)	46 (69.7)	35 (62.5)	37 (58.7)	31 (52.5)	28 (47.5)	31 (48.4)	19 (31.1)	21 (34.4)
TSAT <20% or ferritin <100 ng/mL	20 (28.2)	19 (35.8)	20 (30.3)	21 (37.5)	26 (41.3)	28 (47.5)	31 (52.5)	33 (51.6)	42 (68.9)	40 (65.6)

Systolic blood pressure, mm Hg, mean (SD)	137.09 (15.16)	142.15 (16.11)	135.48 (13.69)	137.45 (13.52)	135.95 (16.92)	136.27 (13.0)	136.32 (15.79)	134.77 (14.45)	141.26 (14.28)	137.73 (16.40)
Diastolic blood pressure, mm Hg, mean (SD)	75.57 (8.93)	76.63 (9.42)	75.32 (8.71)	77.93 (10.17)	73.10 (11.28)	76.41 (10.39)	74.20 (10.23)	72.50 (12.11)	76.04 (10.78)	73.31 (8.89)

hsCRP quintile cut-off was based on patients with non-missing baseline hsCRP values.

eGFR, estimated glomerular filtration rate; Hb, hemoglobin; hsCRP, high-sensitivity C-reactive protein; MedDRA, Medical Dictionary for Regulatory Activities; NDD, non–dialysis-dependent; Q, quintile; Rox, roxadustat; TSAT, transferrin saturation.

[a]Age at study entry.

[b]Medical history events in the MedDRA version 10.0 high-level term diabetes mellitus (including subtypes).

Table S3. Baseline demographics and disease characteristics in the DD population by hsCRP quintile

	hsCRP Q1 ≤1.40 mg/L		hsCRP Q2 >1.40 – ≤2.97 mg/L		hsCRP Q3 >2.97 – ≤5.98 mg/L		hsCRP Q4 >5.98 – ≤13.55 mg/L		hsCRP Q5 >13.55 mg/L	
	**Roxadustat n=405**	**ESA n=413**	**Roxadustat n=411**	**ESA n=402**	**Roxadustat n=373**	**ESA n=440**	**Roxadustat n=433**	**ESA n=381**	**Roxadustat n=400**	**ESA n=414**
Age,[a] y, mean (SD)	52.6 (15.7)	52.8 (15.4)	56.3 (15.2)	56.8 (14.3)	56.9 (14.3)	56.6 (14.5)	57.3 (14.6)	58.1 (14.1)	56.8 (14.1)	58.6 (13.8)
Gender, male, n (%)	227 (56.0)	209 (50.6)	243 (59.1)	234 (58.2)	208 (55.8)	252 (57.3)	250 (57.7)	234 (61.4)	246 (61.5)	266 (64.3)
Race, n (%)										
White	267 (65.9)	276 (66.8)	289 (70.3)	277 (68.9)	275 (73.7)	313 (71.1)	294 (67.9)	260 (68.2)	291 (72.8)	298 (72.0)
Black or African American	47 (11.6)	47 (11.4)	55 (13.4)	63 (15.7)	51 (13.7)	69 (15.7)	76 (17.6)	68 (17.8)	69 (17.3)	63 (15.2)
Asian	70 (17.3)	74 (17.9)	41 (10.0)	42 (10.4)	34 (9.1)	43 (9.8)	37 (8.5)	31 (8.1)	24 (6.0)	27 (6.5)
Native Hawaiian or other Pacific Islander	2 (0.5)	0 (0)	0 (0)	2 (0.5)	1 (0.3)	0 (0)	0 (0)	0 (0)	1 (0.3)	2 (0.5)
American Indian or Alaska native	11 (2.7)	8 (1.9)	10 (2.4)	9 (2.2)	3 (0.8)	6 (1.4)	3 (0.7)	8 (2.1)	6 (1.5)	14 (3.4)
Other	8 (2.0)	8 (1.9)	16 (3.9)	9 (2.2)	9 (2.4)	9 (2.0)	23 (5.3)	14 (3.7)	9 (2.3)	10 (2.4)
Body mass index, kg/m^2, mean (SD)	23.9 (4.15)	24.6 (4.81)	26.5 (5.52)	26.8 (5.20)	28.0 (5.91)	27.5 (6.06)	29.4 (6.88)	29.1 (6.88)	29.8 (7.52)	30.0 (7.84)
Hb, g/dL, mean (SD)	9.72 (1.43)	9.75 (1.34)	9.78 (1.27)	9.92 (1.23)	9.80 (1.32)	9.90 (1.23)	9.90 (1.18)	9.96 (1.23)	9.79 (1.23)	9.62 (1.38)
Ferritin, µg/L, mean (SD)	540.4 (416.53)	533.7 (414.91)	553.2 (433.47)	577.0 (451.72)	588.9 (430.91)	654.2 (468.60)	655.0 (448.59)	690.3 (528.21)	744.2 (519.85)	694.2 (540.50)
Albumin, g/L, mean (SD)	38.6 (4.10)	38.4 (4.14)	38.2 (3.89)	38.0 (3.75)	37.9 (3.53)	38.0 (3.55)	37.2 (3.62)	37.1 (3.26)	36.0 (4.02)	35.8 (4.48)
Cardiovascular disease history, yes, n (%)	146 (36.0)	147 (35.6)	162 (39.4)	180 (44.8)	160 (42.9)	186 (42.3)	215 (49.7)	187 (49.1)	188 (47.0)	201 (48.6)
Type of dialysis,[b] n (%)										
Hemodialysis	350 (86.4)	357 (86.4)	379 (92.2)	369 (91.8)	345 (92.5)	412 (93.6)	406 (93.8)	352 (92.4)	354 (88.5)	384 (92.8)
Peritoneal dialysis	54 (13.3)	56 (13.6)	32 (7.8)	33 (8.2)	28 (7.5)	28 (6.4)	26 (6.0)	29 (7.6)	46 (11.5)	30 (7.2)
Diabetes,[c] n (%)	148 (36.5)	159 (38.5)	160 (38.9)	173 (43.0)	162 (43.4)	187 (42.5)	191 (44.1)	181 (47.5)	197 (49.3)	209 (50.5)
Iron status, n (%)										
TSAT ≥20% and ferritin ≥100 ng/mL	366 (90.4)	378 (91.5)	366 (89.1)	360 (89.6)	327 (87.7)	405 (92.0)	381 (88.0)	330 (86.6)	319 (79.8)	316 (76.3)
TSAT <20% or ferritin <100 ng/mL	39 (9.6)	35 (8.5)	43 (10.5)	40 (10.0)	46 (12.3)	35 (8.0)	52 (12.0)	50 (13.1)	81 (20.3)	97 (23.4)

Systolic BP, mm Hg, mean (SD)	139.81 (17.15)	139.93 (17.58)	140.62 (17.23)	141.33 (18.65)	142.65 (17.41)	140.46 (17.67)	140.71 (19.06)	141.18 (16.65)	140.92 (17.79)	140.01 (18.28)
Diastolic BP, mm Hg, mean (SD)	78.19 (10.68)	77.82 (10.92)	77.67 (10.62)	77.24 (11.23)	77.71 (9.92)	77.20 (10.48)	77.31 (10.83)	76.40 (9.98)	76.71 (10.04)	76.76 (9.90)

hsCRP quintile cut-off is based on patients with non-missing baseline hsCRP values.

BP, blood pressure; DD, dialysis-dependent; ESA, erythropoiesis-stimulating agent; Hb, hemoglobin; hsCRP, high-sensitivity C-reactive protein; MedDRA, Medical Dictionary for Regulatory Activities; Q, quintile; TSAT, transferrin saturation.

[a]Age at study entry.

[b]One patient was missing in the hsCRP Q1 roxadustat treatment group and one patient was missing in the hsCRP Q4 roxadustat treatment group.

[c]Medical history events in the MedDRA version 10.0 high-level term diabetes mellitus (including subtypes).

Table S4. Safety in the overall NDD and DD populations

	NDD population		DD population	
**n (%),<br>no. of events**	**Roxadustat<br>n=323**	**ESA<br>n=293**	**Roxadustat<br>n=2358**	**ESA<br>n=2368**
TEAE	296 (91.6)<br>2730	271 (92.5)<br>2498	2039 (86.5)<br>18,252	2030 (85.7)<br>18,752
Drug-related TEAE[a]	78 (24.1)<br>144	66 (22.5)<br>122	289 (12.3)<br>539	143 (6.0)<br>197
Serious TEAE[b]	209 (64.7)<br>630	181 (61.8)<br>580	1288 (54.6)<br>3932	1260 (53.2)<br>4007
TEAE leading to death	34 (10.5)<br>42	34 (11.6)<br>46	359 (15.2)<br>380	359 (15.2)<br>376
Death (overall)[c]	40 (12.4)	37 (12.6)	492 (20.9)	429 (18.1)

DD, dialysis-dependent; ESA, erythropoiesis-stimulating agent; n, number of patients; no., number; NDD, non–dialysis-dependent; TEAE, treatment-emergent adverse event.

[a]Possible or probable, as assessed by the investigator, or records where relationship is missing.
[b]Includes serious AEs upgraded by the sponsor based on a review of the sponsor's list of "Always Serious Terms" if any upgrade was done.
[c]All reported deaths after the study's first drug administration.

Figure S1. Mean TSAT change from baseline to weeks 28–52 in the (A) NDD patient population[a,b] and the (B) DD patient population[c,d]

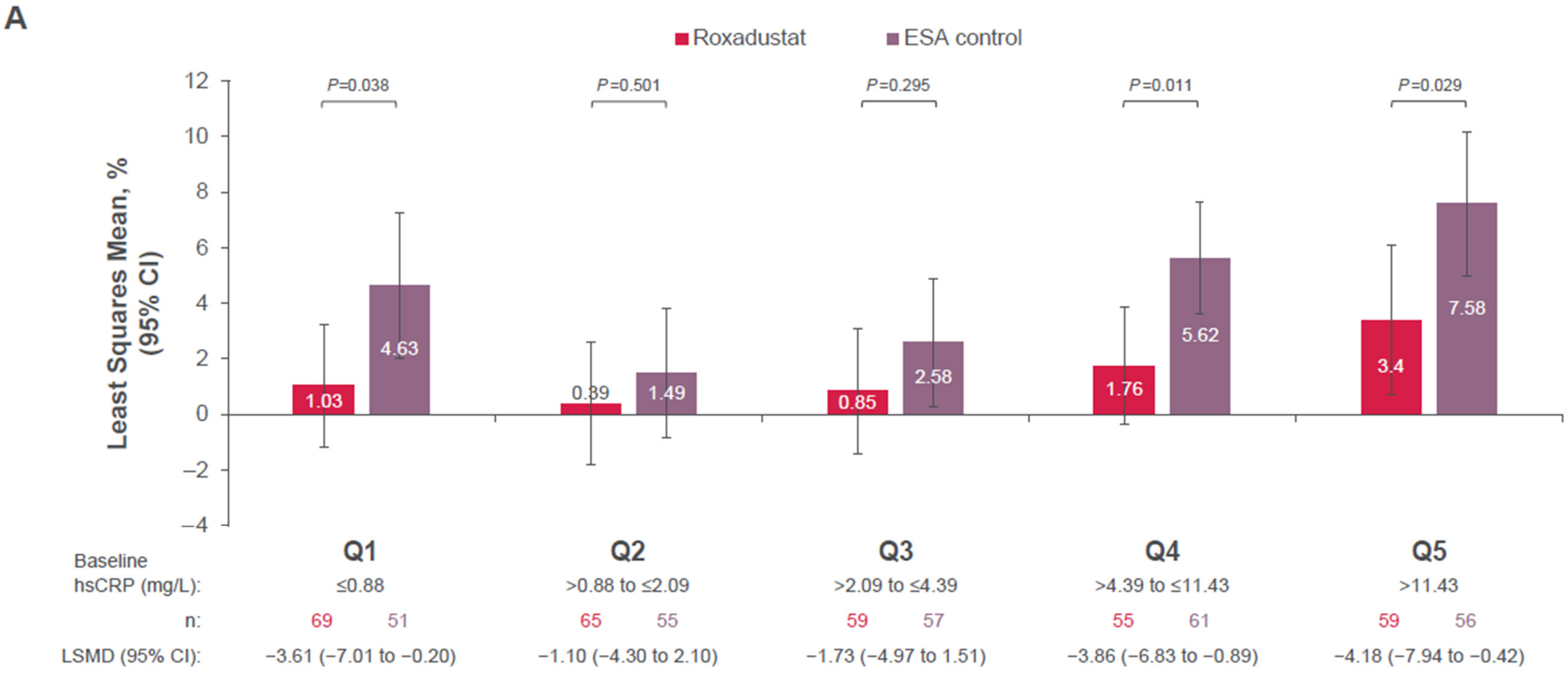

B

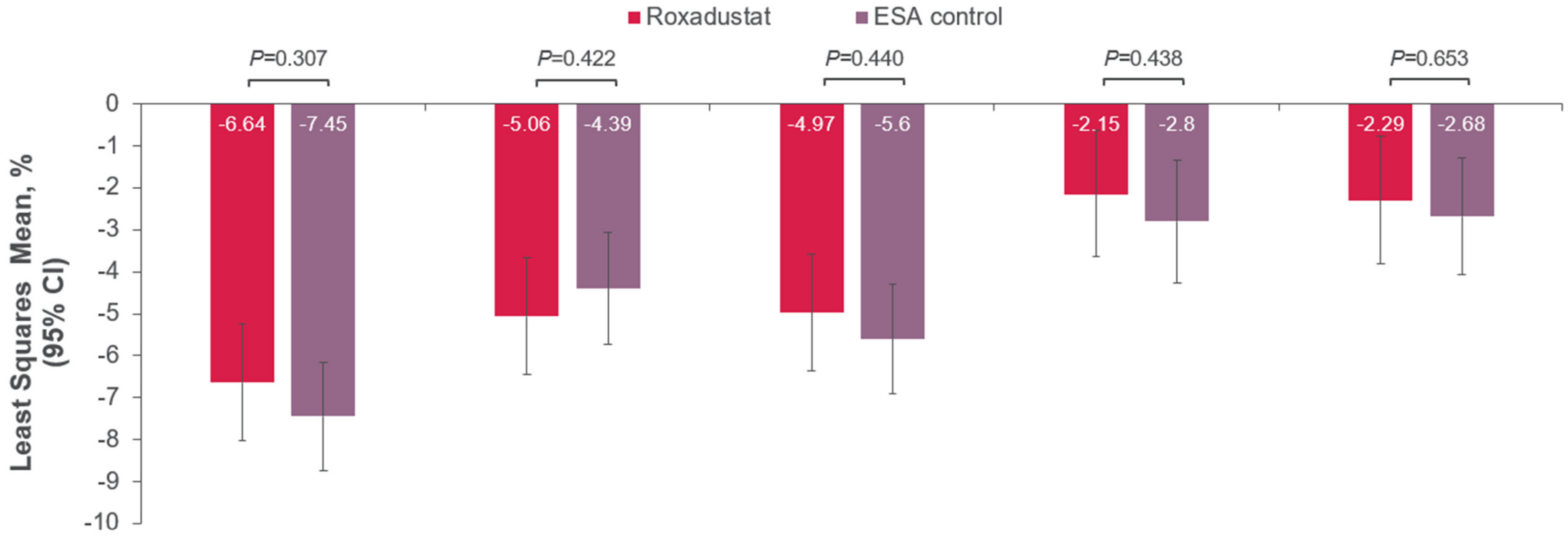

	Q1	Q2	Q3	Q4	Q5
Baseline hsCRP (mg/L):	≤1.40	>1.40 to ≤2.97	>2.97 to ≤5.98	>5.98 to ≤13.55	>13.55
n:	390 404	389 389	347 407	398 353	349 368
LSMD (95% CI):	0.81 (−0.75 to 2.37)	−0.67 (−2.30 to 0.96)	0.63 (−0.96 to 2.22)	0.65 (−0.99 to 2.30)	0.39 (−1.32 to 2.10)

ANCOVA, analysis of covariance; CI, confidence interval; DD, dialysis-dependent; eGFR, estimated glomerular filtration rate; ESA, erythropoiesis-stimulating agent; hsCRP, high-sensitivity C-reactive protein; LS, least squares; LSMD, least squares mean difference; MAR, missing at random; NDD, non-dialysis-dependent; Q, quintile; TSAT, transferrin saturation.

[a]Change in TSAT from baseline to mean during Weeks 28 to 52 was analyzed using an ANCOVA model with the following fixed effects covariates at baseline: TSAT and eGFR values in continuous scales and treatment group.

[b]Adjusted LS means, their difference and corresponding CIs were generated from datasets where missing data were imputed using MAR-based multiple imputation by treatment group, with baseline TSAT and baseline eGFR as predictor variables.

[c]Change in TSAT from baseline to mean during Weeks 28 to 52 was analyzed using an ANCOVA model with baseline TSAT as the covariate and cardiovascular/cerebrovascular/thromboembolic history, geographical region, incident vs. stable dialysis (≤4 months vs >4 months, respectively), and treatment groups as fixed effects.

[d]Adjusted LS means, their difference and corresponding CIs were generated from datasets where missing data were imputed using MAR-based multiple imputation by treatment group, with baseline TSAT, cardiovascular/cerebrovascular/thromboembolic history, geographical region, and incident vs. stable dialysis (≤4 months vs. >4 months, respectively) as predictor variables.
